# Biochemical Characterization and Crystal Structure of a Novel NAD^+^-Dependent Isocitrate Dehydrogenase from *Phaeodactylum tricornutum*

**DOI:** 10.3390/ijms21165915

**Published:** 2020-08-18

**Authors:** Shi-Ping Huang, Lu-Chun Zhou, Bin Wen, Peng Wang, Guo-Ping Zhu

**Affiliations:** Anhui Provincial Key Laboratory of Molecular Enzymology and Mechanism of Major Diseases and Key Laboratory of Biomedicine in Gene Diseases and Health of Anhui Higher Education Institutes, College of Life Sciences, Anhui Normal University, Wuhu 241000, China; huangsp2016@ahnu.edu.cn (S.-P.H.); zlc001@ahnu.edu.cn (L.-C.Z.); wenbin1991@ahnu.edu.cn (B.W.)

**Keywords:** *Phaeodactylum tricornutum*, isocitrate dehydrogenase, biochemical characterization, kinetics, crystal structure

## Abstract

The marine diatom *Phaeodactylum tricornutum* originated from a series of secondary symbiotic events and has been used as a model organism for studying diatom biology. A novel type II homodimeric isocitrate dehydrogenase from *P. tricornutum* (PtIDH1) was expressed, purified, and identified in detail through enzymatic characterization. Kinetic analysis showed that PtIDH1 is NAD^+^-dependent and has no detectable activity with NADP^+^. The catalytic efficiency of PtIDH1 for NAD^+^ is 0.16 μM^−1^·s^−1^ and 0.09 μM^−1^·s^−1^ in the presence of Mn^2+^ and Mg^2+^, respectively. Unlike other bacterial homodimeric NAD-IDHs, PtIDH1 activity was allosterically regulated by the isocitrate. Furthermore, the dimeric structure of PtIDH1 was determined at 2.8 Å resolution, and each subunit was resolved into four domains, similar to the eukaryotic homodimeric NADP-IDH in the type II subfamily. Interestingly, a unique and novel C-terminal EF-hand domain was first defined in PtIDH1. Deletion of this domain disrupted the intact dimeric structure and activity. Mutation of the four Ca^2+^-binding sites in the EF-hand significantly reduced the calcium tolerance of PtIDH1. Thus, we suggest that the EF-hand domain could be involved in the dimerization and Ca^2+^-coordination of PtIDH1. The current report, on the first structure of type II eukaryotic NAD-IDH, provides new information for further investigation of the evolution of the IDH family.

## 1. Introduction

Isocitrate dehydrogenase (IDH) plays a critical role in the tricarboxylic acid (TCA) cycle and catalyzes the oxidative decarboxylation of isocitrate to generate CO_2_ and α-ketoglutarate (α-KG) with divalent metal ions (Mg^2+^ or Mn^2+^) while reducing NAD(P)^+^ to NAD(P)H [1]. In accordance with the preference of coenzyme, IDHs are divided into two forms: NAD^+^-dependent IDH (EC 1.1.1.41, NAD-IDH) and NADP^+^-dependent IDH (EC 1.1.1.42, NADP-IDH). NAD-IDH catalyzes the formation of NADH, which provides electrons for ATP production and participates in energy metabolism [2]. NADP-IDH provides reducing power for biosynthesis by generating NADPH [3]. Meanwhile, NADP-IDH is pivotal in cellular antioxidation damage and reactive oxygen species (ROS) detoxification systems [4,5,6]. Most recently, human mitochondrial and cytoplasmic NADP-IDH mutations have been frequently reported in a variety of cancers, including malignant gliomas and acute myeloid leukemia (AML). The mutated NADP-IDHs acquire a novel activity of catalyzing the reduction of α-KG to D-2-hydroxyglutarate (D2HG), which causes histone methylation reprogramming and promotes tumorigenesis [7,8,9].

IDHs are widely distributed in living organisms, including bacteria, archaea, and eukaryotes. A phylogenetic analysis revealed that the IDH protein family can be divided into three subfamilies: type I, type II, and type III [10,11]. All archaeal and the vast majority of bacterial homodimeric NAD(P)-IDHs and eukaryotic hetero oligomeric NAD-IDHs are grouped together in the type I subfamily. Eukaryotic mitochondrial or cytoplasmic homodimeric NADP-IDHs and a few bacterial NAD(P)-IDHs are categorized into the type II subfamily. Some homodimeric NAD-IDHs from marine bacteria and algae are also clustered into the type II subfamily [11,12]. In contrast to the type I and II IDHs with 400-amino acid polypeptide chains, the bacterial monomeric NAD(P)-IDHs belonged to the type III subfamily, the polypeptide chains of which are approximate 740-amino acid in length. We demonstrated in a previous study that some monomeric IDHs exist as dimers in solution, thus expanding the diversity of the IDH family [10].

Homodimeric NADP-IDHs of prokaryotes and eukaryotes have been extensively studied, such as the type I NADP-IDH from *Escherichia coli* (EcIDH) and the type II NADP-IDH from *Homo sapiens* (HcIDH) [13,14,15,16]. Although the sequence identities between the type I and type II NADP-IDHs are low (<15%), their three-dimensional structures have similar folds, and the key amino acid residues for catalytic activity are conserved. However, information about homodimeric NAD-IDHs is scarce. So far, there is only one type I homodimeric NAD-IDH structure in the Protein Data Bank, which comes from *Acidithiobacillus thiooxidans* (AtIDH) [17]. Structural comparisons have revealed that AtIDH shares the greatest structural similarity to type I NADP-IDHs, but has many differences from the type II NADP-IDHs [16,18,19]. We previously characterized the enzymology and molecular evolutionary mechanisms of some type II homodimeric NAD-IDHs, but their structures have not yet been determined [11,12]. Due to the lack of detailed structural information, the differences in the catalytic mechanism between eukaryotic and prokaryotic homodimeric NAD-IDH are currently still unclear.

*Phaeodactylum tricornutum* is a unicellular marine model diatom and an important primary producer. Benefiting from a fast growth rate and high lipid productivity, *P. tricornutum* has been shown to be an excellent candidate for biodiesel production [20,21]. The genome and expressed sequenced tags (ESTs) analysis have revealed that *P. tricornutum* contains hundreds of genes transferred from bacteria [22,23]. Interestingly, genomic analysis of *P. tricornutum* showed that it encodes two IDHs, one a putative NAD-IDH and the other a putative NADP-IDH. Other unicellular marine algal species, e.g., *Ostreococcus tauri* and *Micromonas* sp., possess only one *idh* gene encoding homodimeric NAD-IDH [12]. When compared to *O*. *tauri* (12.5 Mb), *P. tricornutum* has a larger genome (27.4 Mb), suggesting that the ancestor of the diatom might have obtained many genes from other species for adapting to complex ecological environments [24,25].

In the present study, we investigated the overexpression, purification, and detailed biochemical characteristics of a type II homodimeric NAD-IDH from *P. tricornutum* (PtIDH1). In addition, we presented a substrate/coenzyme-free structure of PtIDH1 (PtIDH1-Apo) based on X-ray diffraction to a resolution of 2.80 Å. To our knowledge, PtIDH1 is the first eukaryotic homodimeric NAD-IDH structure proposed in the type II subfamily, and it exhibits a unique EF-hand domain at the C-terminal. Furthermore, the structure of PtIDH1 was compared with other IDH structures from *H. sapiens* (PDB entry: 1T0L) and *A. thiooxidans* (PDB entry: 2D4V). These results not only provide basic knowledge of eukaryotic homodimeric NAD-IDH but also refine the phylogeny and structural information of the IDH family.

## 2. Results

### 2.1. Sequence Analysis

**Figure 1:**
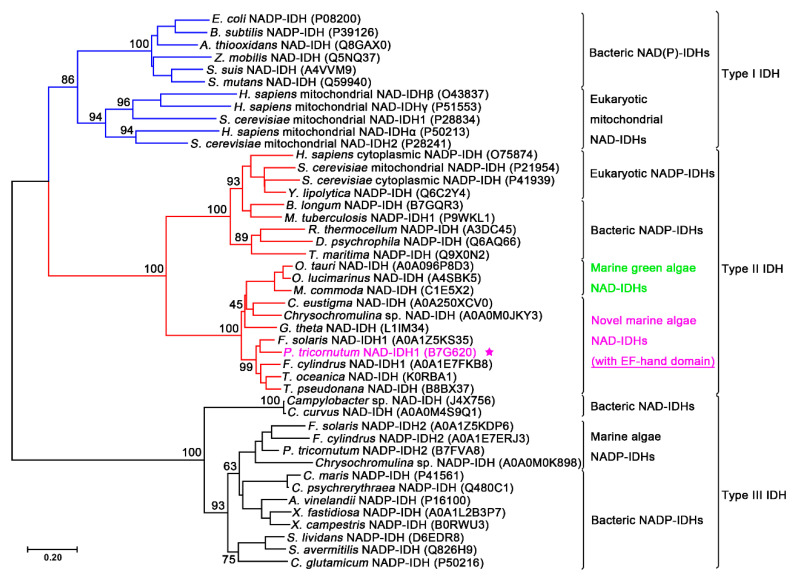
Phylogenetic analysis of IDHs from different species. The analysis involved 45 IDH sequences and a neighbor-joining tree with 1000 bootstraps and was created by MEGA 7.0. The UniProt entry numbers are noted in parentheses. PtIDH1 is marked by a purple star.

The *PtIDH1* gene (Gene ID: Phatr3_J14762) consists of two exons and one intron with a total length of 1740 bp, and the coding sequence (CDS) encodes a 548-amino acid polypeptide (pre-PtIDH1). The amino acid sequence of PtIDH1 showed a high sequence identity to marine algae IDH, such as *Fistulifera solaris* IDH (78%), *Thalassiosira pseudonana* IDH (67%), and *O. tauri* IDH (61%). However, PtIDH1 shared low homology with prokaryotic IDHs, including *Mycobacterium tuberculosis* IDH (23%) and *A. thiooxidans* IDH (11%). Previous investigations redefined the type II IDH subfamily by adding a novel eukaryotic homodimeric NAD-IDH subgroup, which may be an ancestral form of the type II subfamily [12]. A phylogenetic tree was reconstructed to further illuminate the evolutionary relationship between PtIDH1 and other IDHs. The results clearly indicated that PtIDH1 clustered with several homodimeric NAD-IDHs from marine algae, such as *O. tauri* IDH and *M. commoda* IDH [11,12], suggesting that PtIDH1 is a member of the type II NAD-IDHs subfamily (Figure 1).

**Figure 2:**
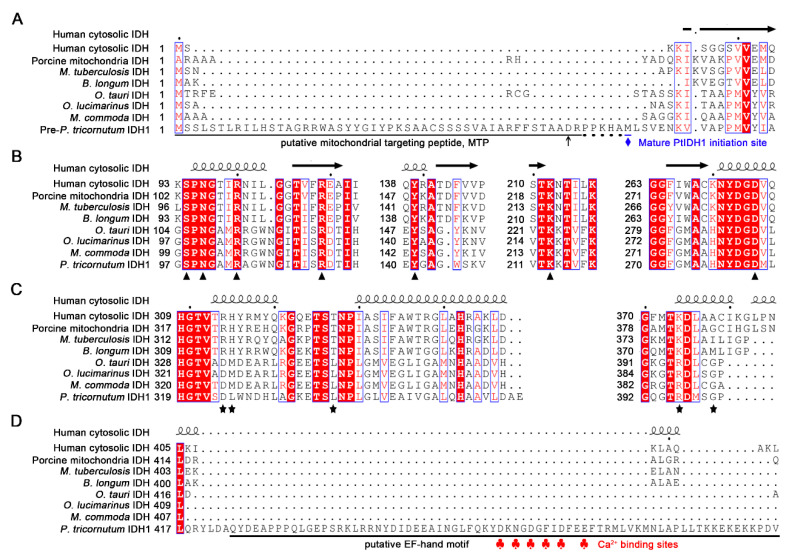
Structure-based amino acid sequence alignment of PtIDH1 with other IDHs. (**A**) Comparison of the N-terminal amino acid sequences of PtIDH1 with other IDHs. The putative mitochondrial targeting peptide sequences are underlined in black, and the possible cleavage sites are indicated by dots. (**B**) The substrate and metal ion binding conserved amino acid residues of the IDH family are indicated by triangles. (**C**) The putative conserved residues implicated in PtIDH1 coenzyme binding are compared with other homomeric NAD(P)-IDHs. The residues that directly or indirectly interact with the 2′-phosphate of NADP^+^ are indicated by stars. (**D**) Comparison of the C-terminal amino acid sequence of PtIDH1 with other IDHs. The putative EF-hand domain sequence is underlined in black. The Ca^2+^-binding sites are indicated by red clubs. The secondary structures of human cytosolic NADP-IDH (PDB entry: 1T0L) was placed above the alignment.

Furthermore, phylogenetic analysis revealed that pre-PtIDH1 contained a predicted 53-amino acid putative mitochondrial-targeting peptide (MTP) at the N-terminal, hinting that PtIDH1 might be targeted to the mitochondria (Figure 2A). To evaluate the potential substrate binding sites and coenzyme preference of PtIDH1, structure-based multiple amino acid sequence alignments were performed (Figure 2B,C). The results indicated that all residues involved in substrate binding were completely conserved in both NAD^+^- and NADP^+^-dependent IDHs of the type II subfamily; however, the putative amino acid residue determinants in coenzyme preference were significantly dissimilar. Two critical residues, Arg and His, which form hydrogen bonds with the 2′-phosphate group of NADP^+^ in NADP-IDHs, were replaced by Asp and Met/Leu in NAD-IDHs. Meanwhile, Asp324 in PtIDH1 was equivalent to Asp344 in *O. tauri* NAD-IDH (OtIDH), which plays a decisive role in NAD^+^ specificity, implying that PtIDH1 could be an NAD^+^-dependent enzyme [12]. Additionally, a unique motif was observed at the C-terminal domain (approximately 70 amino acids) of PtIDH1, and a conserved domain search revealed that it was an EF-hand motif (Figure 2D). There was no related domain in all previously reported IDHs, suggesting that PtIDH1 may possess a special structure and function.

### 2.2. Overexpression and Purification of PtIDH1

**Figure 3:**
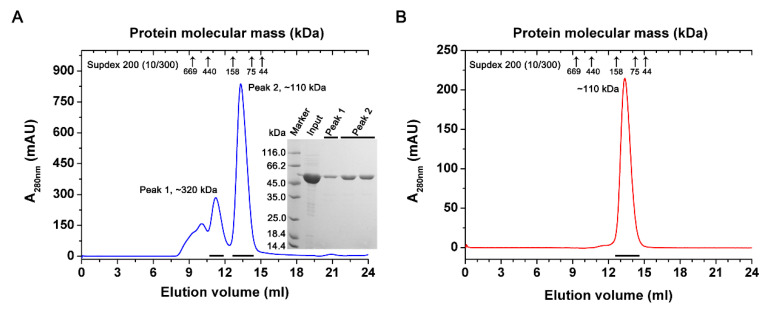
Overexpression, purification, and oligomeric state determination of PtIDH1. (**A**) Gel filtration chromatography elution profile of PtIDH1 from the Superdex 200 (10/300) column. The lower right insert panel shows the protein purity detection by 12% SDS-PAGE. (**B**) Elution profile of dimeric PtIDH1 (Peak 2 from A). All the flow rates of gel filtration chromatography were 0.5 mL·min^−1^.

The mature PtIDH1 (without MTP) was heterologously expressed in *E. coli* Rosetta (DE3) and then purified to homogeneity by Co^2+^ affinity chromatography. SDS-PAGE analysis revealed that recombinant PtIDH1 with 6×His-tag was approximately 55 kDa, which matches the theoretical value (55.1 kDa) well (Figure 3A). The oligomeric status of recombinant PtIDH1 was determined by gel filtration chromatography. Two major peaks were observed when the native molecular mass of PtIDH1 was calculated, 110 kDa and 320 kDa, indicating that recombinant PtIDH1 could be aggregated into a dimer and hexamer (Figure 3A). The dimeric and hexameric forms could be separated, and further gel filtration chromatography results showed that dimeric PtIDH1 does not re-equilibrate to a heterogenous mixture (Figure 3B). Similar studies have previously been performed in which recombinant IDHs were oligomerized due to the effects of heterologous expression and other factors [12,26,27]. Thus, PtIDH1 was revealed to be a homodimeric enzyme, then concentrated and subjected to various characterization trials.

### 2.3. Kinetics Characterization

The specific activity of the recombinant PtIDH1 with NAD^+^ was determined to be 87.13 ± 0.54 U·mg^−1^ (in the presence of Mn^2+^) and 48.34 ± 4.87 U·mg^−1^ (in the presence of Mg^2+^), and it was not able to catalyze the NADP^+^-linked reactions, indicating that PtIDH1 preferentially uses NAD^+^ as a coenzyme. The kinetic parameters of recombinant PtIDH1 for NAD^+^ are displayed in Table 1. The *K*_m_ values of PtIDH1 for NAD^+^ were 1132.5 ± 23.3 μM with Mn^2+^ and 902.5 ± 59.9 μM with Mg^2+^, and its catalytic efficiency (*k*_cat_/*K*_m_) for NAD^+^ was 0.16 μM^−1^·s^−1^ and 0.09 μM^−1^·s^−1^ in the presence of Mn^2+^ and Mg^2+^, respectively (Figure S1). Compared with other marine algae homodimeric NAD-IDHs, the *k*_cat_/*K*_m_ value for NAD^+^ of PtIDH1 was similar to that of *Micromonas* sp. IDH (0.18 μM^−1^·s^−1^), but lower than those of *O. tauri* IDH (0.43 μM^−1^·s^−1^) and *O. lucimarinus* IDH (0.44 μM^−1^·s^−1^) [12,28]. Moreover, the coenzyme affinity (1/*K*_m_) of PtIDH1 was the lowest among the currently known type II NAD-IDHs.

**Figure 4:**
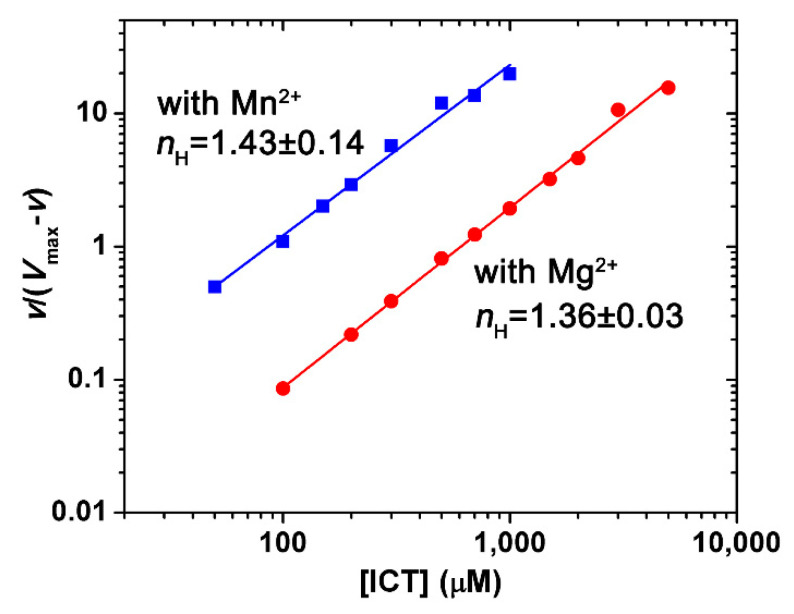
Hill plots of PtIDH1 activity. The *n*_H_ values of PtIDH1 for isocitrate were 1.43 ± 0.14 and 1.36 ± 0.03 in the presence of Mn^2+^ and Mg^2+^, respectively. The mean ± SD values were obtained from three independent replicates.

The substrate kinetic behavior of PtIDH1 was similar to that of *O. tauri* IDH. The double reciprocal plots of the initial reaction rate versus the isocitrate concentration showed a nonlinear relationship. However, Hill plots of the data presented an approximate linear relationship (Figure 4). The *n*_H_ values (Hill coefficient) of PtIDH1 for isocitrate were 1.43 ± 0.14 with Mn^2+^ and 1.36 ± 0.03 with Mg^2+^, demonstrating the existence of cooperative effects. Interestingly, the Hill coefficient of PtIDH1 was comparable to those for *O. tauri* IDH (*n*_H_ = 1.81 with Mn^2+^) and *Chlamydomonas reinhardtii* IDH (*n*_H_ = 1.82 with Mn^2+^) [12,32], but lower than those of eukaryotic hetero oligomeric NAD-IDHs, including *Saccharomyces cerevisiae* mitochondrion NAD-IDH (*n*_H_ = 3.1 with Mg^2+^) [2]. The *S*_0.5_ value of PtIDH1 for isocitrate was 84.9 ± 5.2 μM with Mn^2+^, which was also much lower than potato mitochondrion NAD-IDH (*S*_0.5_ = 690 μM) (Table S1) [33], indicating that PtIDH1 has a high affinity for isocitrate.

### 2.4. Effects of pH and Temperature

**Figure 5:**
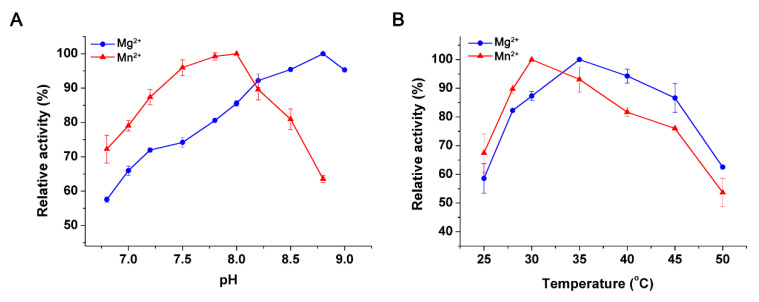

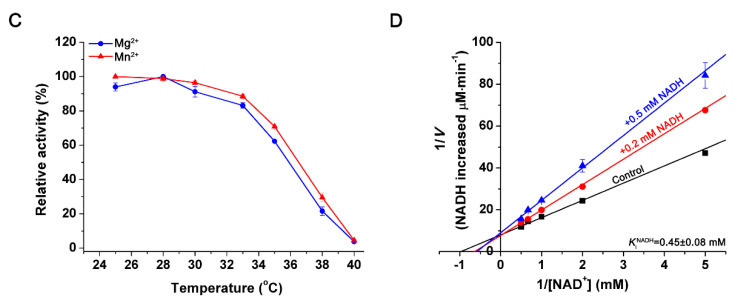
Effect of pH, temperature, and NADH on the NAD^+^-linked activity of PtIDH1. (**A**) Effect of pH on the activity of PtIDH1 in the presence of Mn^2+^ and Mg^2+^, respectively. (**B**) Effect of temperature on the activity of PtIDH1 in the presence of Mn^2+^ and Mg^2+^. (**C**) Heat-inactivation profiles of PtIDH1 in the presence of Mn^2+^ and Mg^2+^. (**D**) Effect of NADH on the activity of PtIDH1 with Mn^2+^. All values are displayed as the means of at least three independent measurements.

The optimal pH values of the recombinant PtIDH1 were 8.0 with Mn^2+^ and 8.8 with Mg^2+^ (Figure 5A), similar to eukaryotic homodimeric *O. tauri* NAD-IDH (pH 8.0 with Mn^2+^) [12,28], but higher than bacterial homodimeric NAD(P)-IDHs, such as *Leptospira interrogans* NADP-IDH (pH 7.0 with Mn^2+^), *Bifidobacterium longum* NADP-IDH (pH 7.5 with Mn^2+^), and *Streptococcus suis* NAD-IDH (pH 7.0 with Mn^2+^) [34,35,36]. Even so, the optimal pH of PtIDH1 was lower than that of bacterial monomeric IDH, including *Corynebacterium glutamicum* IDH (pH 9.0 with Mg^2+^) and *Streptomyces avermitilis* IDH (pH 9.4 with Mn^2+^) [37,38].

PtIDH1 exhibited maximal activity around 30 °C and 35 °C in the presence of Mn^2+^ and Mg^2+^, respectively (Figure 5B). The optimal temperature values were similar to marine bacterial NAD-IDH from *Congregibacter litoralis* (35 °C with Mn^2+^ or Mg^2+^), but apparently lower than marine algae NAD-IDHs from *O. tauri* (45 °C with Mn^2+^ or Mg^2+^), *O. lucimarinus* (40 °C with Mn^2+^ or Mg^2+^), and *C. reinhardtii* (40 °C with Mn^2+^) [11,12,28,32]. Heat inactivation profiles suggested that PtIDH1 was stable below 28 °C, but rapidly lost activity above 30 °C, and only 60% activity remained after a 20-min incubation at 35 °C (Figure 5C).

### 2.5. Effects of Metal Ions and Metabolites

The effects of nine metal ions on the activity of recombinant PtIDH1 were measured in the NAD^+^-linked reaction (Table 2). The results revealed that PtIDH1 was entirely dependent on the presence of a divalent cation, similar to all other previously characterized IDHs. The most effective activator for PtIDH1 catalysis was Mn^2+^, followed by Mg^2+^, which can partially replace the activation of Mn^2+^ (59%). Except for Mn^2+^ and Mg^2+^, no monovalent metal cations (Na^+^, K^+^, and Li^+^) and divalent metal cations could be used as an activator for PtIDH1, and Co^2+^, Cu^2+^, and Ni^2+^ inhibited the activity of PtIDH1 in the presence of Mn^2+^ or Mg^2+^. Generally, Ca^2+^ showed the greatest inhibitory effects on the activity of previously reported IDHs, but it did not inactivate PtIDH1.

In addition, NADH was a competitive inhibitor of PtIDH1 (*K*_i_ = 0.45 ± 0.08 mM) (Figure 5D), which is consistent with *O. tauri* NAD-IDH (*K*_i_ = 0.14 mM) and *C. reinhardtii* NAD-IDH [12,32]. Although 1.0 mM citrate had almost no effect on the activity of PtIDH1, it inhibited the activity of *C. reinhardtii* NAD-IDH [39]. Excess ATP, ADP, AMP, and α-KG (>0.1 mM) strongly inhibited the activity of PtIDH1, as was also observed for *O. tauri* NAD-IDH and *C. reinhardtii* NAD-IDH (Table 3) [12,39].

### 2.6. Crystal Structure of PtIDH1

**Figure 6:**
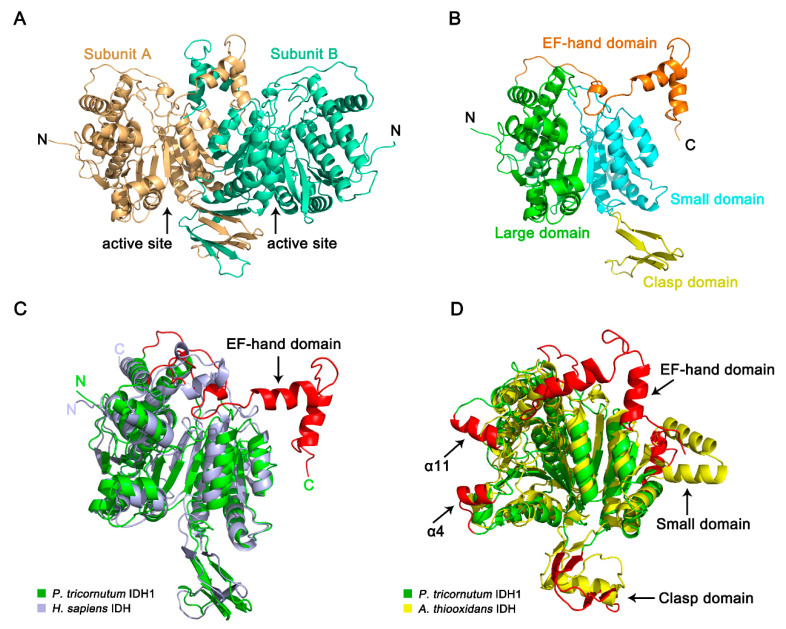
The structural characterizations of PtIDH1. (**A**) Overall structure of PtIDH1-Apo. PtIDH1 shows a dimeric structure and contains two subunits, which are colored light orange (subunit A) or spring-green (subunit B). (**B**) View of the monomer of the PtIDH1. Each subunit contains four domains: a large domain, a small domain, a clasp domain, and an EF-hand domain, which are colored green, cyan, yellow, and orange, respectively. (**C**) Monomer comparison of PtIDH1 (green) and HcIDH (light blue) shows the difference in the C-terminus (in red). (**D**) Overlay of the monomer of PtIDH (green) and AtIDH (yellow). The arrows, pointing towards the red ribbon of PtIDH1, represent the structural differences between PtIDH1 and AtIDH.

The apo form crystal structure of PtIDH1 was determined at 2.8 Å resolution in space group P2_1_2_1_2_1_, and the asymmetric unit contained two monomers that form a dimer (Figure 6A, Table S2). The final model was refined to *R*_work_ and *R*_free_ of 22.7% and 28.3%, respectively. Each subunit of PtIDH1 in the final refined structure missed several amino acid residues, including Met1-Val4 and Lys487-Val495 (15 residues). The monomer of PtIDH1 mainly consists of three distinct domains: a large domain, a small domain, and a clasp domain (Figure 6B). The large domain constitutes the 228 residues from both the N- and the C-terminal (Glu5-Asn108 and Ser300-Ala423) and has a typical Rossmann fold. The small domain shows α/β sandwich conformation, with 147 residues (Gly109-Gly142 and Tyr187-Thr299). The clasp domain consists of residues Ala143-Pro186, making up two two-stranded anti-parallel β-sheets. In addition, a unique EF-hand domain is observed at the C-terminal domain of Gln424-Lys486, which is composed of a helix‒loop‒helix conformation. There are no related domains in all previously reported IDHs. As with all structured homodimeric IDHs, there are two clefts on each side of the structure, which are formed by the large and small domains of one subunit and the small domain of the adjacent domain. The putative active sites are located in these two cleft regions.

A structure-similarity search for PtIDH1 was performed by using the DALI online server [40]. The most similar structure was NADP^+^-dependent HcIDH (PDB entry: 1T0L, Z-score of 42.8), and the root-mean-square deviation (RMSD) for the 394 C_α_ atoms of the two structures was 2.2 Å. Then, the monomer of PtIDH1 was superimposed on the structure of HcIDH complexed with isocitrate and NADP^+^. Except for the extra EF-hand domain, mainly secondary structure fold and topology of PtIDH1 were shared similarities with HcIDH (Figure 6C and Figure S2). Furthermore, the PtIDH1 monomer was structurally aligned with AtIDH (PDB entry: 2D4V), a bacterial homodimeric NAD-IDH, yielding a higher RMSD value of 3.3 Å for 344 C_α_ atoms. Despite having highly similar protein folds, structural differences at the large domain and clasp domain were clearly identified (Figure 6D). Initially, the clasp domain in the PtIDH1 structure formed two two-stranded anti-parallel β-sheets, but those of the AtIDH structure formed an α-helix and two anti-parallel β-strands. Additionally, PtIDH1 contained two insertions (Ala84-Gly91 and Ser323-Ala331) in the large domain, specifically forming two α-helices (α4 and α11) at the active site. These characteristic structural differences also exist between other type I and type II homodimeric IDHs and serve as phylogenetic and evolutionary characteristics [16,18].

### 2.7. Mutational Analysis of PtIDH1

**Figure 7:**
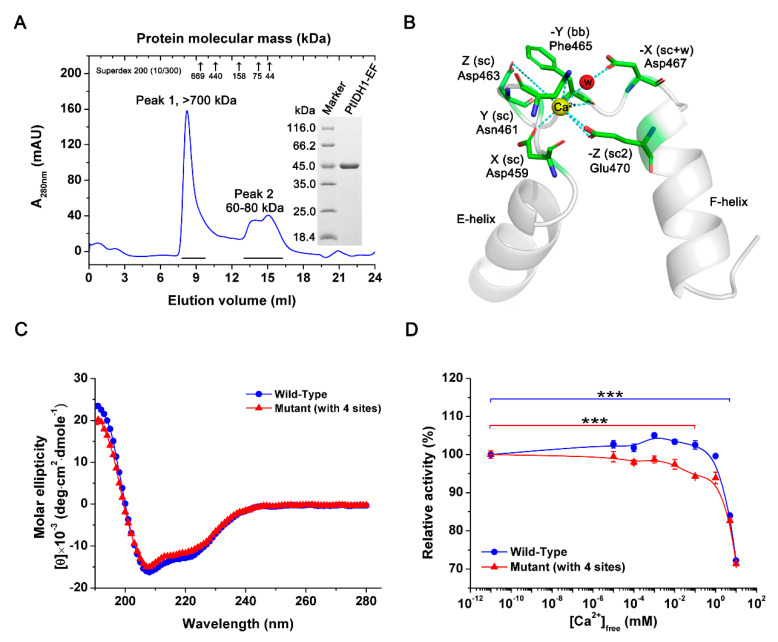
Characterization of the mutants. (**A**) Overexpression and purification of PtIDH1-EF. Gel filtration chromatography elution profile of PtIDH1-EF from the Superdex 200 (10/300) column with a flow rate of 0.5 mL·min^−1^. The inset panel shows the protein purity detection by 12% SDS-PAGE. (**B**) Modeling studies of Ca^2+^ co-ordination by the EF-hand motif of PtIDH1. Ca^2+^ coordinating residues (X, Y, Z, -Y, -Z, and -X) are indicated as sticks and are labeled. The Ca^2+^ ion and the water molecule are indicated in yellow and red, respectively. Side chain, sc; backbone, bb; water molecule, w. (**C**) Circular dichroism (CD) spectra of the wild-type PtIDH1 and its mutant (M4A). (**D**) Effect of Ca^2+^ on the activity of wild-type PtIDH1 and its mutant (M4A). The assay mixtures were prepared as described for a standard reaction in the presence of 2 mM Mn^2+^, and the Ca^2+^ concentration was changed by extra addition. A reaction mixture without added Ca^2+^ was used as a control. *** *p* < 0.001 vs. control.

We investigated the function of the EF-hand domain in the PtIDH1 structure by site-directed mutagenesis. The mutant without the EF-hand domain (PtIDH1-EF, deleted Gln424-Val495 in a total of 72 residues) was successfully overexpressed in *E. coli* cells and purified by the previously described protocol. The SDS-PAGE analysis results indicated a single band of about 45 kDa for PtIDH1-EF, consistent with the calculated value (about 46.5 kDa) (Figure 7A). However, the enzyme assays showed that PtIDH1-EF completely lost its catalytic activity. Elution on gel filtration chromatography suggested that the dimeric association of the wild-type enzyme was disrupted, resulting in aggregation of PtIDH1-EF in solution (Figure 7A). Moreover, the dimeric interface area of PtIDH1 (4082 Å^2^) was larger than that of HcIDH (3638 Å^2^), and at least a 1700 Å^2^ interface area was provided by the EF-hand domain of PtIDH1 at the C-terminal (Figure S3). Therefore, the dimerization was formed through the unique C-terminal EF-hand domain in PtIDH1.

The EF-hand domain belongs to the calcium-binding protein family and has been known to modulate calcium homeostasis [41]. Generally, the canonical EF-hand loop is composed of twelve residues. The six of Ca^2+^’s seven coordinating bonds are provided by side-chain carboxy groups of several acidic residues, and the other one is provided by a backbone carbonyl group. These coordination effects usually exist in the form of Ca-O bonds. Interestingly, the Ca^2+^-coordinating residues in the loop are conserved, and they are notated based on linear and coordination positions: 1(X), 3(Y), 5(Z), 7(-Y), 9(-X), and 12(-Z) (Figure S4) [42]. The structure reveals that PtIDH1 EF-hand loop contains several residues (Asp459, Asn461, Asp463, Phe465, Asp467, and Glu470) that are aligned well with Ca^2+^-coordinating residues conserved in other EF-hand loops, indicating that PtIDH1 EF-hand loop binds to Ca^2+^ by canonical coordinating mechanism (Figure 7B). Next, we generated two PtIDH1 mutants (M6A: D459A/N461A/D463A/F465A/D467A/E470A and M4A: D459A/N461A/D463A/F465A) in which potential Ca^2+^-coordinating residues in the EF-hand loop were replaced with alanine (Ala, without carboxy side-chains) via site-directed mutagenesis. Unfortunately, the M6A mutant was not sufficiently expressed in the *E. coli* expression system; only the M4A mutant was overexpressed and purified. The circular dichroism (CD) spectra and calculated secondary structure contents of M4A mutant (31.1 ± 0.5% α-Helix, 18.1 ± 0.1% β-Strand, 12.5 ± 0% Turn and 34.0 ± 0.2% Random) were very similar to the wild-type enzyme (32.1 ± 1.5% α-Helix, 17.3 ± 0.5% β-Strand, 12.5 ± 0% Turn and 33.6 ± 0.5% Random), which demonstrated that mutations in PtIDH1 did not dramatically change the mainly conformation (Figure 7C). Enzyme assays showed that M4A maintained comparable specific activity (78.0 ± 1.0 U·mg^−1^) to that of wild-type PtIDH1 (87.1 ± 0.54 U·mg^−1^). Next, we evaluated the effect of Ca^2+^ on PtIDH1 activity under the addition of increasing concentrations of CaCl_2_ (from 0 mM to 10 mM) to the assay mixture in the presence of Mn^2+^. The wild-type PtIDH1 exhibited a strong calcium tolerance and maintained its original activity under 2 mM Ca^2+^. On the contrary, as the Ca^2+^ concentrations increased, the activity of the M4A mutant rapidly decreased; this was extremely significant at 0.1 mM Ca^2+^ (Figure 7D).

## 3. Discussion

IDHs are important participants in energy metabolism and biosynthesis and are indispensable in all organisms. Eukaryotic NAD-IDHs, as the key rate-limiting enzymes in the TCA cycle, are almost entirely located in the mitochondria [43]. Enzymatic characterization has revealed that recombinant PtIDH1 is a completely NAD^+^-dependent IDH and exhibits allosteric regulation by isocitrate, similar to eukaryotic mitochondrial NAD-IDHs from *O. tauri*, *S. cerevisiae*, *Pisum sativum*, and *Solanum tuberosum* [2,12,33,44]. Therefore, PtIDH1 with the N-terminal targeting peptide sequence is very likely a mitochondrial enzyme.

Early phylogenetic analysis and competition experiments have demonstrated that NAD^+^ dependence is an ancestral trait, NADP^+^ dependence by bacterial IDHs is an adaptive trait, and the alteration of the coenzyme specificity is an adaptive result [45]. For IDHs of the type I subfamily, the coenzyme specificity can be completely converted from NAD^+^ to NADP^+^, such as *Xylella fastidiosa* NAD-IDH and *Pyrococcus furiosus* NAD-IDH [31,46], or from NADP^+^ to NAD^+^, such as *E. coli* NADP-IDH [47]. Interestingly, the evolutionary mechanism may be common for all members of type II IDHs. Our previous studies also successfully converted the coenzyme specificity of type II IDH from NAD^+^ to NADP^+^, including *O. tauri* NAD-IDH and *O. lucimarinus* NAD-IDH [11,12], or from NADP^+^ to NAD^+^, such as *Bifidobacterium longum* NADP-IDH [35]. Secondary structure-based sequence alignment revealed that the underlying NAD^+^-binding sites of PtIDH1 are Asp324 and Leu325, which are consistent with *O. tauri* NAD-IDH (Asp344 and Met345), but they are substituted by Arg314 and His315 in *H. sapiens* cytoplasmic NADP-IDH (Figure 2). Therefore, the coenzyme specificity of PtIDH1 has the potential to switch from NAD^+^ to NADP^+^ by strategic amino acid replacement, which should be explored in detail in future studies.

Protein structures are usually more conserved than sequences, so structural analysis and comparison are significant to understand protein evolution [48]. For the first time, we reported a novel eukaryotic homodimeric NAD-IDH structure from the marine diatom *P. tricornutum*, expanding the structural information on the IDH protein family. A structural comparison revealed that the crystal structure of PtIDH1 was different from the reported bacterial NAD-IDH in the type I subfamily and shared the highest structural similarity to NADP-IDHs in the type II subfamily, demonstrating that PtIDH1 is an important member of type II and providing strong evidence that PtIDH1 and its counterparts are possible ancestors of NADP-IDHs in the type II subfamily. However, there are a few conformational differences in the overall structure between PtIDH1 and type II homodimeric NADP-IDHs. The active site cleft of PtIDH1 presents an open conformation, and the distance of the active site entrance is 24.5 Å (the distance between residues 80 and 257), which is comparable with that in the open form of HcIDH (PDB entry: 1T09, the distance between its equivalents residues 76 and 250 is 21.2 Å) (Figure S5) [16]. Moreover, an extra EF-hand domain covers the NAD^+^ binding sites and may hinder the entry of coenzymes to the active site in PtIDH1. These special conformations could explain why PtIDH1 has such a low affinity for NAD^+^, which resulted in the enzyme’s catalytic efficiency being significantly lower than that of other homologs. These results indicate that the catalytic mechanism of PtIDH1 is more complex than that of other type II IDHs.

The EF-hand domain belongs to the superfamily of calmodulin (CaM), occurring widely in proteins from different organisms, such as bacteria, plants, and animals [49]. Many environmental factors can cause the flow of Ca^2+^ between the cytoplasm and subcellular organelles, to act as the secondary messenger [50]. As a Ca^2+^ sensor and adaptor, the EF-hand domain participates in many cellular processes, including signal transduction and calcium homeostasis [41]. Although Ca^2+^ plays a fundamental signaling role in almost all organisms, it seems to be incompatible with IDH. Generally, Ca^2+^ is considered to be an inhibitor of IDH. The quaternary complex structure of *E. coli* IDH (EcIDH-isocitrate-NADP^+^-Ca^2+^) provides evidence that isocitrate-Ca^2+^ can bind to the enzyme, causing the inhibition of EcIDH activation [51]. However, enzymatic functional studies have confirmed that PtIDH1 can coordinate calcium, and the EF-hand domain is pivotal in this process. The existence of the C-terminal EF-hand domain of PtIDH1, a potential mitochondrial enzyme, implies that mitochondria may be an important storage “pool” of Ca^2+^ in *P. tricornutum*.

Furthermore, we used PtIDH1 as a query to search its homologs by using the BLAST program in NCBI. The results showed that the distribution of IDHs with a C-terminal extra EF-hand domain is very limited: only a few species of marine algae possess this type of IDH (Figure 1). It is worth noting that these marine algae all belong to the Stramenopiles lineage, which has experienced several endosymbiotic events and contains a secondary plastid [52]. A recent study indicated that the EF-hand domain from Stamenopiles has a bacterial origin, and the evolutionary relationships are very close to those from *Acidobacteria* [53]. Thus, this special combination of structures of PtIDH1 is presumed to be caused by horizontal gene transfer (HGT).

In summary, we have reported the detailed biochemical characteristics of a novel eukaryotic homodimeric NAD-IDH and determined the crystal structure. The ancient phenotype and conserved structure reveal that PtIDH1 and its counterparts, the putative homodimeric NAD-IDHs from marine algae, are possibly the ancestors of type II subfamily. This finding expands our evolutionary information on the IDH protein family. Further structures of the PtIDH1 complex with NAD^+^, isocitrate, and metal ions, however, are needed to better understand the catalytic and regulatory molecular mechanism of this novel type II homodimeric NAD-IDH.

## 4. Materials and Methods

### 4.1. Strains and Cultivation

The *P. tricornutum* strain (FACHB-2174) was purchased from the Freshwater Algae Culture Collection at the Institute of Hydrobiology, Chinese Academy of Sciences (FACHB, Wuhan, China). Microalgae cells were grown in 100 mL Erdschreiber’s liquid medium (FACHB, Wuhan, China), modified after the original Plymouth seawater recipe, at 22 ± 1 °C and under 100 μmoL photons m^−2^ s^−1^, following a 12 h:12 h light/dark photoperiod. The *E. coli* TOP10 and Rosetta (DE3) were stored at an Ultra-low temperature freezer in our laboratory.

### 4.2. Sequence Analysis

Based on the *P. tricornutum* genome database Ensembl Protists website (http://protists.ensembl.org/Phaeodactylum_tricornutum/Info/Index/), the full-length gene coding region of *P. tricornutum* IDH1 (Gene ID: Phatr3_J14762) was downloaded. The *P. tricornutum* IDH1 signal peptide and subcellular localization prediction were inspected with SignaIP v. 5.0 web server (http://www.cbs.dtu.dk/services/SignalP/), TargetP v. 2.0 server (http://www.cbs.dtu.dk/services/TargetP/), and WoLF PSORT (https://wolfpsort.hgc.jp/) [54,55,56]. Protein sequence similarity and identity between *P. tricornutum* IDH1 and other IDHs were analyzed with BLASTP (https://blast.ncbi.nlm.nih.gov/Blast.cgi). The X-ray crystallographic structures of IDHs were downloaded from the Protein Data Bank database (https://www.rcsb.org/). Structure-based multiple amino acid sequence alignment was performed with T-Coffee servers (http://tcoffee.crg.cat/) and the ESPript 3.0 web server (http://espript.ibcp.fr/ESPript/cgi-bin/ESPript.cgi) [57,58]. Phylogenetic analysis by the neighbor-joining method with 1000 bootstraps was created by the MEGA 7.0 program [59]. Diagrams of the protein structure were drawn by PyMOL software [60].

### 4.3. PtIDH1 Gene Cloning and Plasmid Construction

For RNA isolation, the *P. tricornutum* cells were harvested by centrifugation at 1500× *g* and 4 °C for 10 min. Total RNA of *P. tricornutum* was extracted using the RNAprep Pure Plant Kit (Cat.#DP432, TIANGEN Biotech, Beijing, China) according to the user’s manual. The first-strand cDNA was synthesized from 2 ng of total RNA with TransScript II First-Strand cDNA Synthesis SuperMix (Cat.#AH301-02, TransGen Biotech, Beijing, China) according to the manuals.

According to the coding sequence of IDH1 from the genome of *P. tricornutum* CCAP1055/1, two pairs of primers (Table S3) were designed to amplify the full-length *PtIDH1* and shortened (without the sequence encoding MTP, residues 1‒53) genes. Amplification using a polymerase chain reaction (PCR) program was performed as follows: 95 °C for 3 min, 30 cycles of 95 °C for 30 s, 55 °C for 15 s, and 72 °C for 25 s. The PCR products with *Nde*I and *Xho*I (Thermo Scientific, Shanghai, China) digestion were cloned into expression vector pET-22b(+). The correct recombinant plasmids, verified by sequencing (General Biosystems, Hefei, China), were introduced into the prokaryotic expression strain *E. coli* Rosetta (DE3).

### 4.4. Site-Directed Mutagenesis

In order to verify the functionality of the EF-hand domain in PtIDH1, one truncation (PtIDH1-EF, without the EF-hand domain) and two mutations were constructed by site-directed mutagenesis. The primers for creating the mutants are presented in Table S3. The pET-*PtIDH1-EF* recombinant plasmid was constructed based on a previously described protocol. Two mutant genes were obtained by the overlap extension PCR technique. Taking M4A as an example, the upstream and downstream fragments with the desired mutation sites were obtained by the first PCR step, using the primer pair PtIDH1-53_S& PtIDH1_M4A_As and Mutant_As& PtIDH1_M4A_S. The two overlapping fragments were joined by a fusion PCR program as follows: 95 °C for 3 min, 5 cycles of 95 °C for 30 s, 68 °C for 30 s, and 72 °C for 25 s. Then, the full-length mutant genes were amplified with primers PtIDH1-53_S and Mutant_As. After digestion with *Nde*I and *Xho*I, the final PCR products were ligated to the expression vector pET-22b to create the recombinant plasmid pET-M4A. Similar methods were used for the pET-M6A with recombinant plasmid pET-M4A as the template. All mutated genes were identified by sequencing (General Biosystems, Hefei, China).

### 4.5. Recombinant Protein Overexpression and Purification

*E. coli* Rosetta (DE3) cells bearing recombinant expression plasmid DNA were cultured overnight at 37 °C in 5 mL Luria–Bertani (LB) medium containing 100 µg·mL^−1^ ampicillin and 30 µg·mL^−1^ chloramphenicol. Next, 2000 mL of LB medium in a shake flask, supplemented with the same antibiotics, was inoculated with 2 mL of the overnight culture and incubated at 37 °C. When the cell density at A600 (OD_600_) reached 0.6–0.8, isopropyl-β-D-thiogalactopyranoside (IPTG) was added to the cultures so that the final concentration was 0.4 mM. Culturing continued for 20 h at 18 °C. Then, the cells were collected by centrifugation at 5000× *g* for 8 min at 4 °C. The harvested cells were resuspended and sonicated in 30 mL Lysis buffer (20 mM Tris, 300 mM NaCl and 5% Glycerol at pH 7.5) and then centrifuged at 12,000× *g* for 30 min at 4 °C. Finally, the recombinant target protein with 6×His-tagged was purified by a Co^2+^ affinity resin (Cat.#635502, Clontech, TaKaRa, Dalin, China) column according to the user’s manual. The purity and molecular mass of PtIDH1 were analyzed by 12% SDS–polyacrylamide gel electrophoresis (SDS–PAGE).

### 4.6. Gel Filtration Chromatography

Gel filtration chromatography was performed with the ÄKTA purifier protein purification system (GE Healthcare Life Sciences, Pittsburgh, PA, USA). A GE Filtration Calibration Kit HMW was used to calibrate the Superdex 200 10/300 increase column (GE Healthcare Life Sciences, Pittsburgh, PA, USA), and a standard curve was drawn based on five standard proteins: Thyroglobulin (669 kDa), Ferritin (440 kDa), Aldolase (158 kDa), Conalbumin (75 kDa), and Ovalbumin (44 kDa). All samples were centrifuged at 4 °C and 12,000× *g* for 20 min to remove the precipitated materials and soluble gases that were stored in the equilibration buffer (20 mM Tris, 300 mM NaCl, and 5% Glycerol at pH 7.5).

### 4.7. Circular Dichroism Spectroscopy

Circular dichroism (CD) spectra were detected using a Jasco model J-810 spectropolarimeter instrument. PtIDH1 and mutant protein concentrations were diluted to 0.2 mg·mL^−1^ using a CD buffer that included 20 mM NaH_2_PO_4_, 75 mM Na_2_SO_4_, and 5% glycerol at pH 7.5. Then, 200 µl of the protein was placed in a microcuvette that was transferred to the instrument at a wavelength range of 190 nm to 280 nm. The mean residue ellipticity ([*θ*], deg·cm^2^·dmole^−1^) of the protein was calculated according to the following formula [*θ*] = *θ*/10·(*n* − 1)·*C*·l, where *θ* is the ellipticity measured in mdeg; *n* is the number of protein amino acid residues; *C* is the concentration of the protein in mol/L; and l is the light path of the cuvette (0.1 cm). For each sample, three scans were performed and the average taken. Estimation of the protein secondary structure method was described by Raussens et al. [61].

### 4.8. Enzyme Assays and Kinetic Characterization

The enzyme assays were conducted in a 1-mL reaction system containing 50 mM Tris at pH 8.0, 2 mM MnCl_2_ or MgCl_2_, 1 mM DL-isocitric acid trisodium, and 2 mM NAD^+^ or 5 mM NADP^+^ at 25 °C. The increase in NAD(P)H (ε_340_ = 6.22 mM^−1^·cm^−1^) was determined at 340 nm by a Cary 300 UV-Vis spectrophotometer (Agilent, Santa Clara, CA, USA). The enzyme activity was calculated by taking the average value of 2‒3 parallel tests. The protein concentrations were determined by a Quick Start Bradford Protein Assay kit (Cat.#500-0207, Bio-Rad, Hercules, CA, USA). One unit of enzyme activity refers to 1 μM NAD(P)H produced per minute.

The kinetic parameters of PtIDH1 were determined by measuring the enzyme activity at variable concentrations of DL-isocitrate or NAD^+^. The *K*_m_ and *k*_cat_ values for NAD^+^ were calculated by nonlinear fitting using GraphPad Prism 7.0 (San Diego, CA, USA). The Hill coefficients (*n*_H_) and *S*_0.5_ values for DL-isocitrate were calculated by fitting the kinetic data to the Hill equation in Origin 8.0 (OriginLab, Northampton, MA, USA). All kinetic parameters were obtained from at least three independent measurements.

The effects of pH, temperature, metal ions, and metabolites on the activity of PtIDH1 were measured by using the standard assay reaction mixture. The optimum pH of PtIDH1 was 6.5–9.0, and the optimum temperature was measured over the range 25–50 °C. The thermostability of recombinant PtIDH1 through heat inactivation was tested by incubating enzyme aliquots at 25–40 °C for 20 min. The effects of 2 mM monovalent metal cations (Na^+^, K^+^, and Li^+^) and divalent metal cations (Mn^2+^, Mg^2+^, Ca^2+^, Co^2+^, Cu^2+^, and Ni^2+^) on PtIDH1 activity were also estimated. The effects of metabolites (NADH, citrate, α-Ketoglutarate, ATP, ADP, and AMP) on recombinant PtIDH1 activity were detected using the standard assay method.

### 4.9. Crystallization and Structure Determination

The recombinant PtIDH1 was further purified by gel filtration chromatography, using a HiLoad Superdex 200 10/60 column (GE Healthcare Life Sciences), and then concentrated to 8 mg/mL for crystal growing. Crystals were obtained by the sitting-drop vapor diffusion method using a crystallization solution containing 200 mM NaCl, 100 mM Sodium HEPES (pH 7.5), and 25% (*w*/*v*) PEG 4000 at 22 °C. Crystals were soaked in the crystallization solution containing 20% (*v*/*v*) glycerol, and then were flash-cooled with liquid nitrogen. During the data collection, the crystal was kept at 100 K. Diffraction images were collected at beam line 18U1 of the Shanghai Synchrotron Radiation Facility (SSRF, Shanghai, China). Diffraction data were processed using the XDS package [62]. The apo form of *O. tauri* IDH (PDB entry: 6IXL, not reported previously) was used as the initial search model for the molecular replacement method by the program PHASER in the CCP4 program suite [63]. Manual model building was performed with COOT [64]. The crystallographic refinements were executed using PHENIX, and the qualities of the final model were evaluated by PROCHECK [65,66]. Finally, the coordinates and structural factors of PtIDH1 were published in the Protein Data Bank under the accession code 6LKZ.

## Figures and Tables

**Table 1:** Comparison of kinetic parameters between PtIDH1 and other NAD-IDHs.

Enzyme	NAD^+^				NADP^+^	
	*K*_m_	*k*_cat_	*k*_cat_/*K*_m_	*K*_m_	*k*_cat_	*k*_cat_/*K*_m_
	(μM)	(s^−1^)	(μM^−1^·s^−1^)	(μM)	(s^−1^)	(μM^−1^·s^−1^)
PtIDH1 (Mn^2+^)	1132.5 ± 23.3	180.5 ± 6.8	0.16	−	−	−
PtIDH1 (Mg^2+^)	903.0 ± 59.9	79.0 ± 1.8	0.09	−	−	−
*O. tauri* IDH [12]	265	115	0.43	4314	46	0.0107
*O. lucimarinus* IDH [11]	136.6	60.3	0.444	2211	10	0.0045
*Micromonas* sp. IDH [11]	126.0	22.5	0.179	1827	1.4	0.0008
*C. litoralis* IDH [28]	309.1	84.7	0.27	−	−	−
*Z. mobilis* IDH [29]	312	88	0.282	8200	14	0.0017
*X. campestris* IDH [30]	225	49	0.213	4322	8.5	0.002
*X. fastidiosa* IDH [31]	121	74.6	0.617	2339	6.1	0.003

−: No detectable activity. Data are the mean ± SD of at least three independent measurements.

**Table 2:** Effect of different metal ions on the activity of PtIDH1.

Metal Ions	Relative Activity (%)	Metal Ions	Relative Activity (%)	Metal Ions	Relative Activity (%)
None	4.83 ± 0.45				
Mn^2+^	100 ± 0.0	Mn^2+^	100 ± 0.0	Mg^2+^	100 ± 0.0
Mg^2+^	59.19 ± 3.40	Mn^2+^ + Mg^2+^	120.09 ± 9.91	Mg^2+^ + Mn^2+^	202.78 ± 3.40
Ca^2+^	3.87 ± 0.43	Mn^2+^ + Ca^2+^	107.11 ± 4.00	Mg^2+^ + Ca^2+^	58.46 ± 2.52
Co^2+^	5.96 ± 0.39	Mn^2+^ + Co^2+^	28.21 ± 1.57	Mg^2+^ + Co^2+^	13.48 ± 1.44
Cu^2+^	5.76 ± 0.86	Mn^2+^ + Cu^2+^	84.94 ± 6.82	Mg^2+^ + Cu^2+^	78.59 ± 1.97
Ni^2+^	1.60 ± 0.36	Mn^2+^ + Ni^2+^	70.58 ± 4.47	Mg^2+^ + Ni^2+^	28.24 ± 0.85
Na^+^	4.66 ± 0.16	Mn^2+^ + Na^+^	114.08 ± 4.93	Mg^2+^ + Na^+^	96.87 ± 3.80
Li^+^	5.15 ± 0.30	Mn^2+^ + Li^+^	109.11 ± 1.88	Mg^2+^ + Li^+^	82.71 ± 5.22
K^+^	4.12 ± 0.52	Mn^2+^ + K^+^	99.07 ± 1.61	Mg^2+^ + K^+^	97.80 ± 5.82

The relative activity was for the standard reaction mixture with the metal ion(s) at 2 mM. Data are the mean ± SD of at least three independent measurements.

**Table 3:** Comparison of metabolites’ effect on PtIDH1, OtIDH, and CrIDH.

Metabolite	Concentration (mM)	Relative Activity (%)		
		PtIDH1	*O. tauri* IDH [12]	*C. reinhardtii* IDH [39]
**control**		100.00 ± 0.0	100	100
**citrate**				
	0.01	101.98 ± 0.9	100.71	100
	0.1	101.47 ± 0.2	103.54	82
	1.0	106.29 ± 0.4	106.45	64
**α-Ketoglutarate**				
	0.01	98.68 ± 1.8	/	/
	0.1	100.69 ± 1.0	100.36	/
	1.0	98.29 ± 0.3	95.49	/
**ATP**				
	0.01	102.06 ± 1.5	93.62	109
	0.1	98.89 ± 0.4	93.62	91
	1.0	88.70 ± 0.8	81.20	54
**ADP**				
	0.01	101.04 ± 2.3	100.99	100
	0.1	97.54 ± 1.5	97.87	109
	1.0	86.63 ± 0.2	95.04	91
**AMP**				
	0.01	102.02 ± 1.7	104.37	109
	0.1	99.74 ± 1.0	99.07	100
	1.0	97.88 ± 4.5	89.61	91

/: Not mentioned. Data are the mean ±SD of at least three independent measurements.

## Supplementary Materials

Supplementary materials can be found at https://www.mdpi.com/1422-0067/21/16/5915/s1.

## Abbreviations

PtIDH1	*Phaeodactylum tricornutum* isocitrate dehydrogenase 1
NAD^+^	β-Nicotinamide adenine dinucleotide
NADP^+^	β-Nicotinamide-adenine dinucleotide phosphate
NAD-IDH	NAD^+^-dependent isocitrate dehydrogenase
NADP-IDH	NADP^+^-dependent isocitrate dehydrogenase
*K*_m_	Michaelis constant
*k*_cat_	catalytic rate constant
*n*_H_	Hill coefficient
CD	circular dichroism
