# Supplementary material for: Biochemical Characterization and Crystal Structure of a Novel NAD+-Dependent Isocitrate Dehydrogenase from Phaeodactylum tricornutum
Source: Int J Mol Sci. 2020 Aug 18;21(16):5915. doi: 10.3390/ijms21165915 (PMC7460673; doi:10.3390/ijms21165915)
Supplement: Supplementary file 1 [file ijms-21-05915-s001.zip › Supplementary_Proof/Proof-supplementary.pdf]

International Journal of *Molecular Sciences*

Biochemical Characterization and Crystal Structure of a Novel NAD+-Dependent Isocitrate Dehydrogenase from *Phaeodactylum tricornutum*

Shi-Ping Huang, Lu-Chun Zhou, Bin Wen, Peng Wang and Guo-Ping Zhu

Supplementary Materials

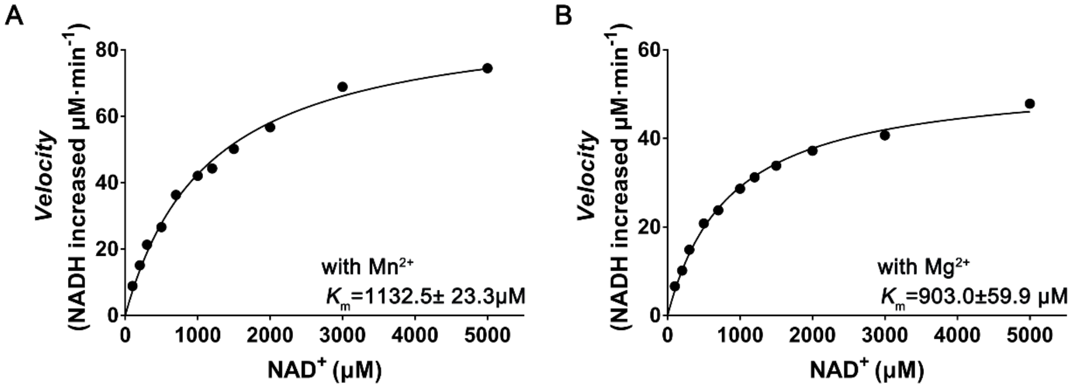

Figure S1. Kinetic analyses of the PtIDH1. The *K*m values of PtIDH1 for NAD^+ were 1132.5 ± 23.3 μM with Mn^{2+} (**A**) and 902.5 ± 59.9 μM with Mg^{2+} (**B**), respectively.

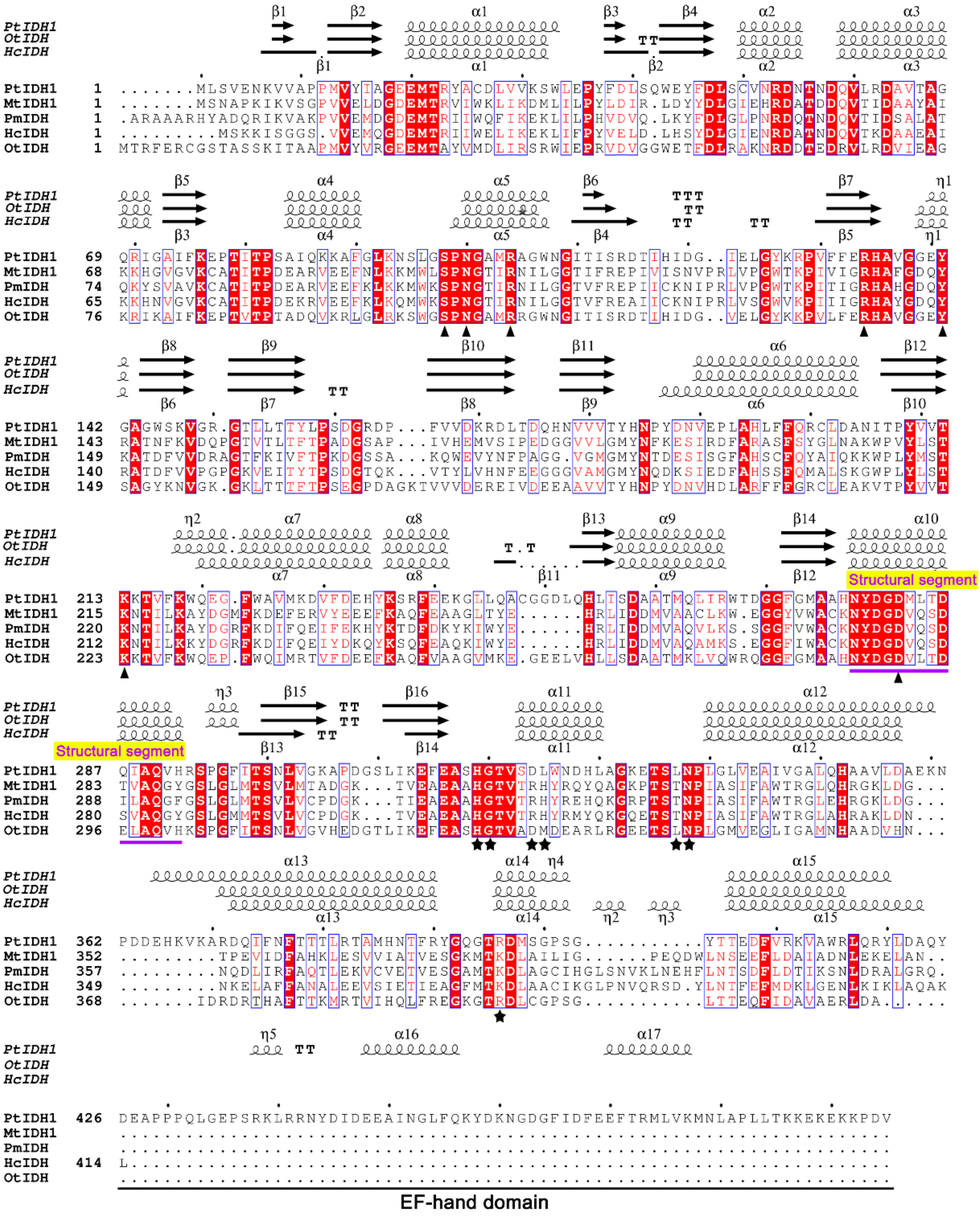

Figure S2. Structure-based multiple sequence alignment of five Type II IDHs. The five IDHs for sequence alignment are PtIDH1, *O. tauri* NAD-IDH (OtIDH), human cytosolic NADP-IDH (HcIDH), porcine mitochondrial NADP-IDH (PmIDH) and *M. tuberculosis* NADP-IDH (MtIDH). The secondary structures of PtIDH1, OtIDH (PDB entry: 6IXN) and HcIDH (PDB entry: 1T0L) are placed above the alignment. The substrate and metal ion binding conserved amino acid residues of the IDH family are indicated by triangles. The residues that directly or indirectly interact with NAD(P)$^+$ are indicated by stars.

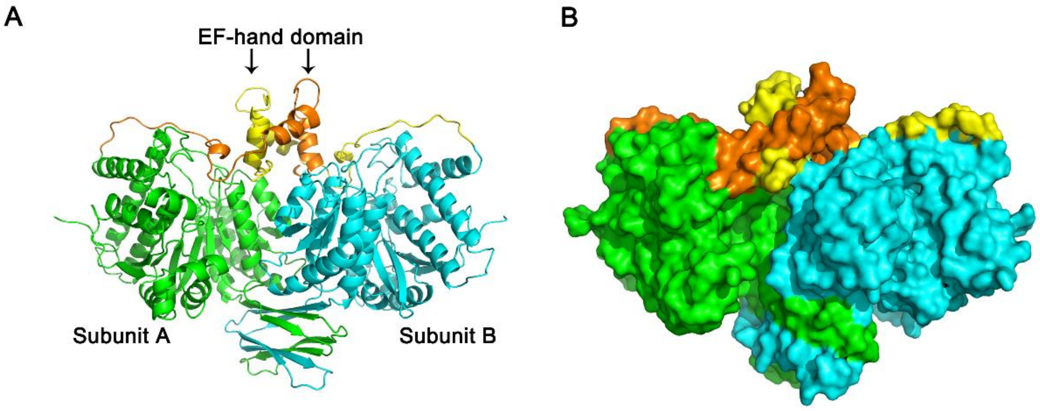

Figure S3. Overall structure of PtIDH1 showed by cartoon (**A**) and surface (**B**), respectively. The small domain, clasp domain and EF-hand domain forming the dimeric interface.

	Ca^{2+}-binding EF-hand loop position											
Coordinating ligand	1<br>X (sc)	2	3<br>Y (sc)	4	5<br>Z (sc)	6	7<br>-Y (bb)	8	9<br>-X (sc+w)	10	11	12<br>-Z (sc2)
Most common	D<br>100%	K<br>29%	D<br>76%	G<br>56%	D<br>52%	G<br>96%	T<br>23%	I<br>68%	D<br>32%	F<br>23%	E<br>29%	E<br>92%
Other frequently observed		AQTVI SER	N	KRN	SN		FKQT ER	VL	STENGQ	YATL EN	DKAP N	D
PtIDH1-EF	D_{459}	K	N	G	D	G	F	I	D	F	E	E_{470}
M4A-EF	A	-	A	-	A	-	A	-	-	-	-	-
M6A-EF	A	-	A	-	A	-	A	-	A	-	-	A

Figure S4. The sequence of EF-hand domain Ca^{2+} binding loop. The Ca^{2+} ligands are indicated with both the linear and coordination positions: 1(X), 3(Y), 5(Z), 7(-Y), 9(-X) and 12(-Z). The percentage of common residues (%) and other frequently observed residues are shown [1]. Side chain, sc; backbone, bb; water molecule, w. The sequence of PtIDH1-EF-hand loop also shown in the figure. M4A-EF: the EF-hand domain of PtIDH1 carries four-site mutant (D459A/D461A/D463A/F465A). M6A-EF: the EF-hand domain of PtIDH1 carries six-site mutant (D459A/D461A/D463A/F465A/D467A/E470A).

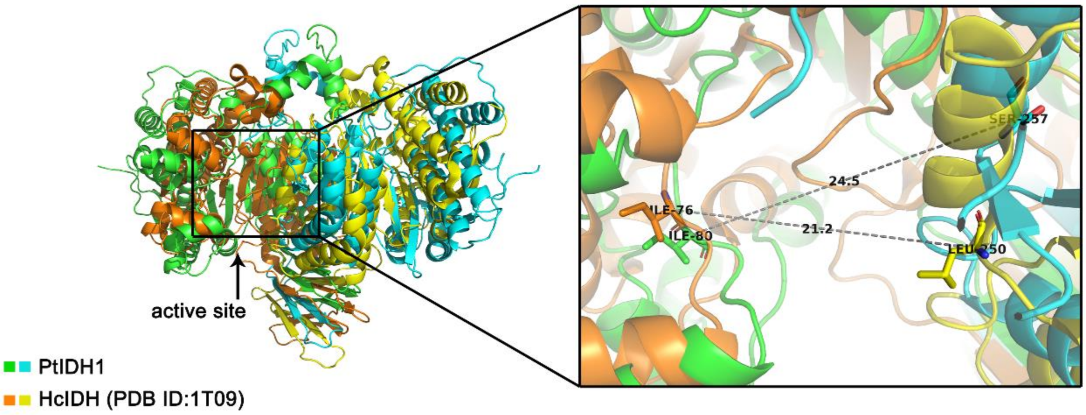

Figure S5. Overlay of the dimers of PtIDH1 (green&cyan) and HcIDH (orange&yellow). The enlarged view (right) highlights the different sizes of active sites between PtIDH1 and HcIDH. The distance between the C_α -atoms of Ile80 and Ser257 in PtIDH1 and Ile76 and Leu250 in HcIDH are indicated.

Table S1. The kinetic parameters of eukaryotic IDHs toward isocitrate.

**Enzyme**	**$S_{0.5}$ (μM)**	**Hill coefficient (n_H)**
PtIDH1 (Mg^{2+})	608.1 ± 4.0	1.36 ± 0.03
PtIDH1 (Mn^{2+})	84.9 ± 5.2	1.43 ± 0.14
O. tauri NAD-IDH (Mg^{2+}) [2]	8.1	1.91
O. tauri NAD-IDH (Mn^{2+}) [2]	6.6	1.83
C. reinhardti NAD-IDH (Mn^{2+}) [3]	370	1.82
S. cerevisiae NAD-IDH (Mg^{2+}) [4]	530	3.1
Pea NAD-IDH (Mg^{2+}) [5]	300	3.1
Potato NAD-IDH (Mg^{2+}) [6]	690	2.5

Data are the mean ± SD of at least three independent measurements.

Table S2. X-ray data collection and structure refinement statistics.

	PtIDH1_Apo
**Data collection**	
X-ray source (Beam line)	SSRF (BL18U1)
Wavelength (Å)	0.97930
Space group	$P\ 2_1\ 2_1\ 2_1$
**Cell dimensions**	
a, b, c (Å)	71.97, 128.55, 124.30
α, β, γ (°)	90.00, 90.00, 90.00
Resolution (Å)	47.94-2.71 (2.86-2.71)
R_{merge} (%) [a]	9.20 (71.60)
I/σI	18.60 (3.80)
Completeness (%)	100.0 (100.0)
No. of observed reflections	422213 (59008)
No. of unique reflections	31982 (4568)
Mosaicity (°)	0.10
Multiplicity	13.20 (12.90)
**Refinement**	
Resolution (Å)	47.04-2.80 (2.90-2.80)
No. of reflections working/test set	30800/1599
R_{work}/R_{free} [b]	0.227/0.283
No. of non-hydrogen atoms (protein/water)	7664/26
**Mean B-factor (Å²)**	
overall	73.88
chain A/B/S	73.01/74.87/54.97
**RMSD from ideal geometry**	
Bond length (Å)	0.01
Bond angles (°)	1.84
**Ramachandran plot**	
Core (%)	84.0
Allowed (%)	13.6
Generously allowed (%)	1.2
Disallowed (%)	1.2
PDB ID	6LKZ

The values in parentheses indicate the highest resolution shell. [a]$R_{merge}=\Sigma_{hkl}\Sigma_i|I_{hkl,i}-\langle I(hkl)\rangle|/\Sigma_{hkl}\Sigma_i I_{hkl}$. $R_{pim}=\Sigma_{hkl}[1/(n-1)]^{1/2}\Sigma_i|I_{hkl,i}-\langle I(hkl)\rangle|/\Sigma_{hkl}\Sigma_i I_{hkl,I}$. [b]$R_{work}=\Sigma||F_{obs}|-|F_{calc}||/\Sigma|F_{obs}|$, where F_{obs} and F_{calc} are the observed and calculated structure factor amplitudes. R_{free} was calculated same as R_{work}, using a randomly selected 5% reflections excluded from refinement.

Table S3. Primers for amplification of wild-type and mutant PtIDH1 genes.

Name [a]	Sequences (5'-3') [b]
PtIDH1_S	GGAATTCCATATGTCCTCCTTGTCAACACTCCGAATC
PtIDH1-53_S	GGAATTCCATATGCTCAGTGTCGAGAATAAAGTCGTG
PtIDH1-EF_As	CCGCTCGAGCGCGTCGAGGTATCGTTGTAAACGCC
PtIDH1_As	CCGCTCGAGCACGTCCGGCTTCTTCTCCTTTTCTTTC
PtIDH1_M4A_S	GTAT***gcc***AAG***gct***GGT***gcc***GGT***gct***ATTGACTTTGAAG
PtIDH1_M4A_As	CTTCAAAGTCAAT***agc***ACC***ggc***ACC***agc***CTT***ggc***ATAC
PtIDH1_M6A_S	GT***gcc***GGT***gct***ATT***gcc***TTTGAA***gca***TTCACTAGAATGC
PtIDH1_M6A_As	GCATTCTAGTGAA***tgc***TTCAAA***ggc***AAT***agc***ACC***ggc***AC
Mutant_As	CTTCCTTTTTCAATATTATTGAAGCATTTATCAGG

[a] "S" and "As": indicate the sense (S) and antisense (As) primers of the corresponding genes. [b] Underlined bases indicate the restriction sites. CATATG, *Nde*I; CTCGAG, *Xho*I. Underlined and bold lower-case bases indicate the mutation site.

References

1. Takahashi, D.; Suzuki, K.; Sakamoto, T.; Iwamoto, T.; Murata, T.; Sakane, F., Crystal structure and calcium-induced conformational changes of diacylglycerol kinase alpha EF-hand domains. *Protein Sci* **2019**, 28, (4), 694-706.
2. Tang, W. G.; Song, P.; Cao, Z. Y.; Wang, P.; Zhu, G. P., A unique homodimeric NAD^+-linked isocitrate dehydrogenase from the smallest autotrophic eukaryote *Ostreococcus tauri*. *FASEB J* **2015,** 29, (6), 2462-72.
3. Martínez-Rivas, J. M.; Vega, J. M., Studies on the isoforms of isocitrate dehydrogenase from *Chlamydomonas reinhardtii*. *J Plant Physiol* **1994,** 143, (2), 129-134.
4. Lin, A. P.; Demeler, B.; Minard, K. I.; Anderson, S. L.; Schirf, V.; Galaleldeen, A.; McAlister-Henn, L., Construction and analyses of tetrameric forms of yeast NAD^+-specific isocitrate dehydrogenase. *Biochemistry* **2011,** 50, (2), 230-9.
5. McIntosh, C. A.; Oliver, D. J., NAD-linked isocitrate dehydrogenase: isolation, purification, and characterization of the protein from pea mitochondria. *Plant Physiol* **1992,** 100, (1), 69-75.
6. Tezuka, T.; Laties, G. G., Isolation and characterization of inner membrane-associated and matrix NAD-specific isocitrate dehydrogenase in potato mitochondria. *Plant Physiol* **1983,** 72, (4), 959-63.
