# Supplementary material for: Biochemical Characterization and Crystal Structure of a Novel NAD+-Dependent Isocitrate Dehydrogenase from Phaeodactylum tricornutum
Source: Int J Mol Sci. 2020 Aug 18;21(16):5915. doi: 10.3390/ijms21165915 (PMC7460673; doi:10.3390/ijms21165915)
Supplement: Supplementary file 1 [file ijms-21-05915-s001.zip › Supplementary_Proof/Proof_Supplementary_ijms-866099.docx]

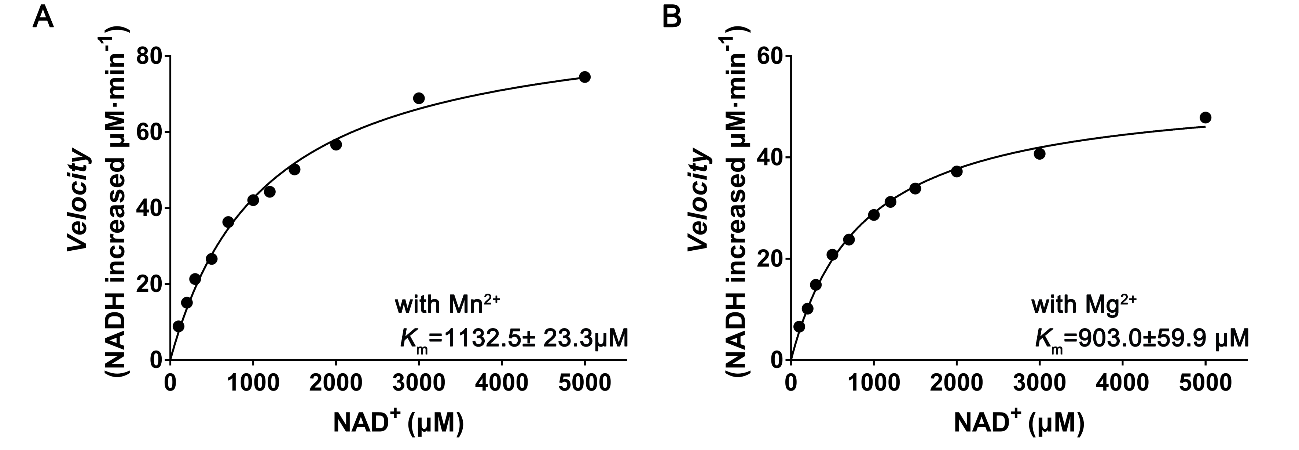


**Figure S1.** Kinetic analyses of the PtIDH1. The *K*_m_ values of PtIDH1 for NAD^+^ were 1132.5 ± 23.3 μM with Mn^2+^ (**A**) and 902.5 ± 59.9 μM with Mg^2+^ (**B**), respectively.


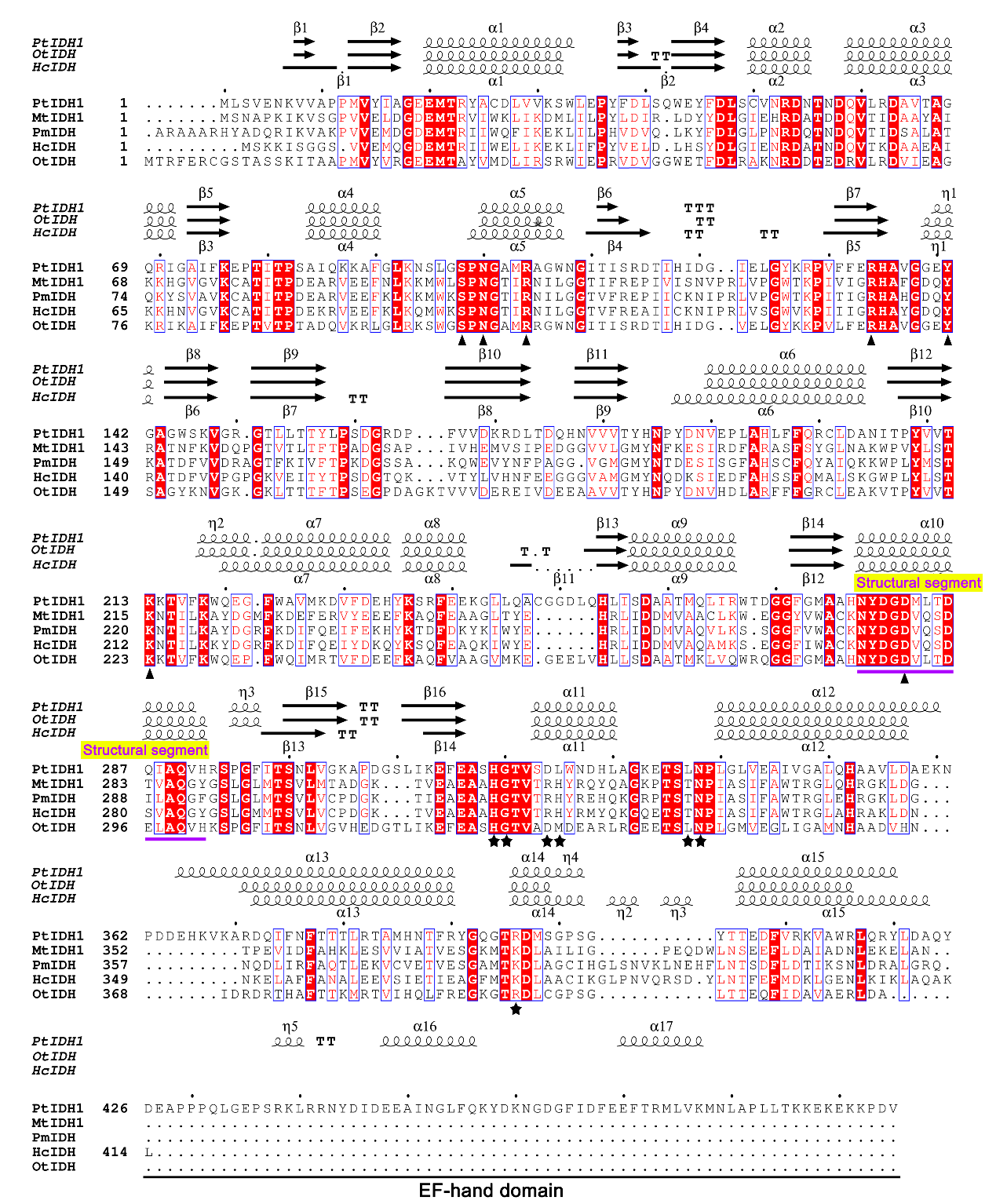


**Figure S2.** Structure-based multiple sequence alignment of five Type II IDHs. The five IDHs for sequence alignment are PtIDH1, *O. tauri* NAD-IDH (OtIDH), human cytosolic NADP-IDH (HcIDH), porcine mitochondrial NADP-IDH (PmIDH) and *M. tuberculosis* NADP-IDH (MtIDH). The secondary structures of PtIDH1, OtIDH (PDB entry: 6IXN) and HcIDH (PDB entry: 1T0L) are placed above the alignment. The substrate and metal ion binding conserved amino acid residues of the IDH family are indicated by triangles. The residues that directly or indirectly interact with NAD(P)^+^ are indicated by stars.


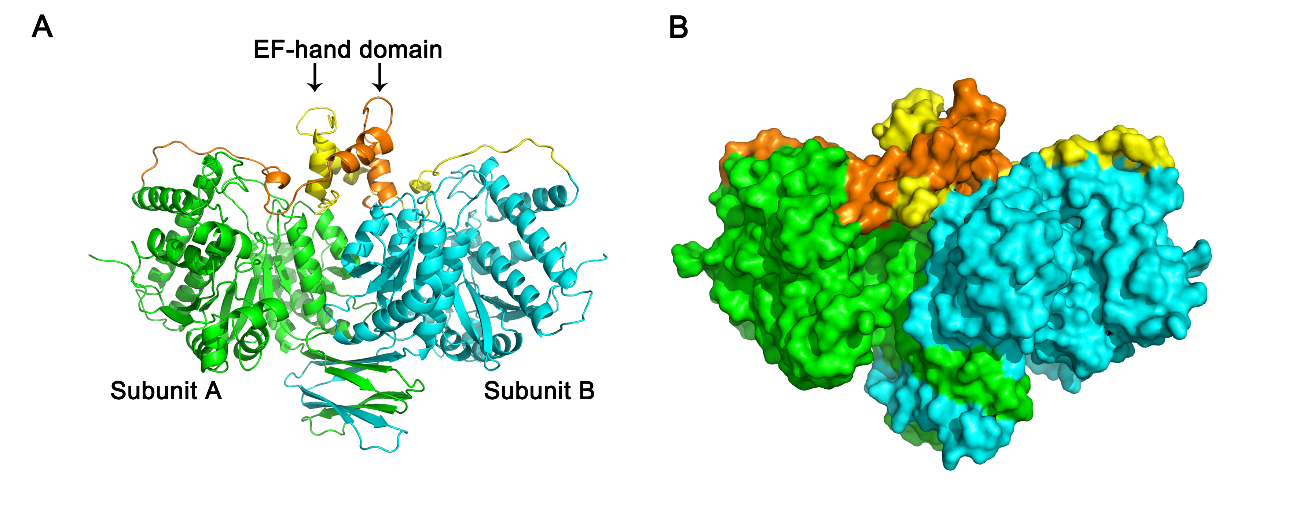


**Figure S3.** Overall structure of PtIDH1 showed by cartoon (**A**) and surface (**B**), respectively. The small domain, clasp domain and EF-hand domain forming the dimeric interface.


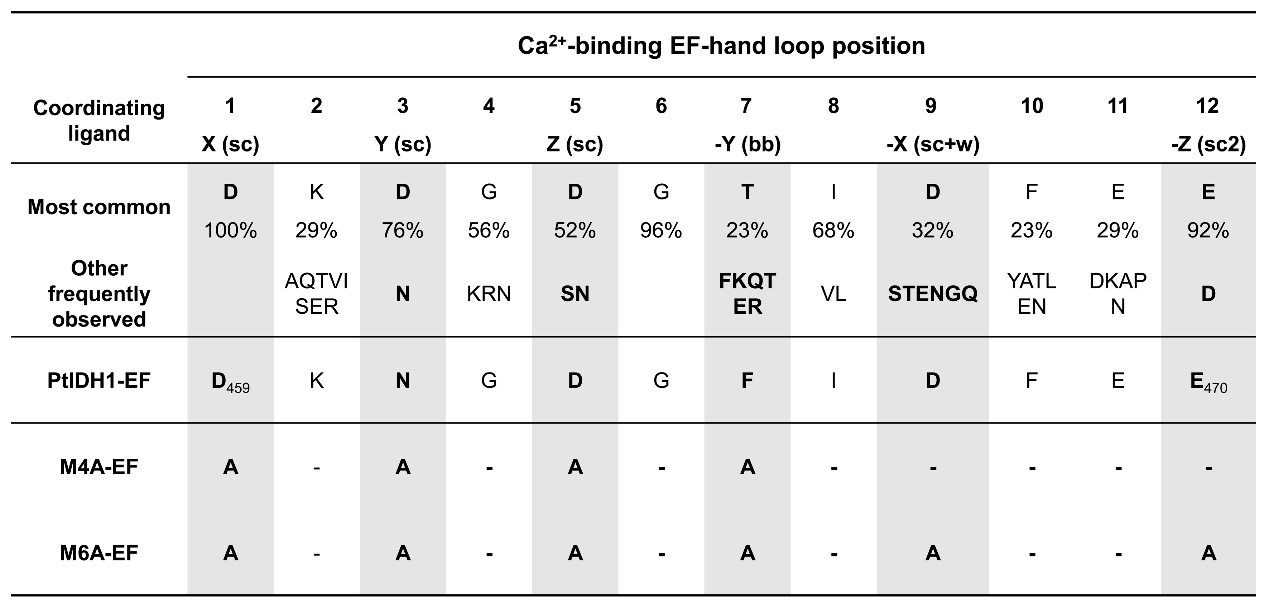


**Figure S4.** The sequence of EF-hand domain Ca^2+^ binding loop. The Ca^2+^ ligands are indicated with both the linear and coordination positions: 1(X), 3(Y), 5(Z), 7(-Y), 9(-X) and 12(-Z). The percentage of common residues (%) and other frequently observed residues are shown [1]. Side chain, sc; backbone, bb; water molecule, w. The sequence of PtIDH1-EF-hand loop also shown in the figure. M4A-EF: the EF-hand domain of PtIDH1 carries four-site mutant (D459A/D461A/D463A/F465A). M6A-EF: the EF-hand domain of PtIDH1 carries six-site mutant (D459A/D461A/D463A/F465A/D467A/E470A).


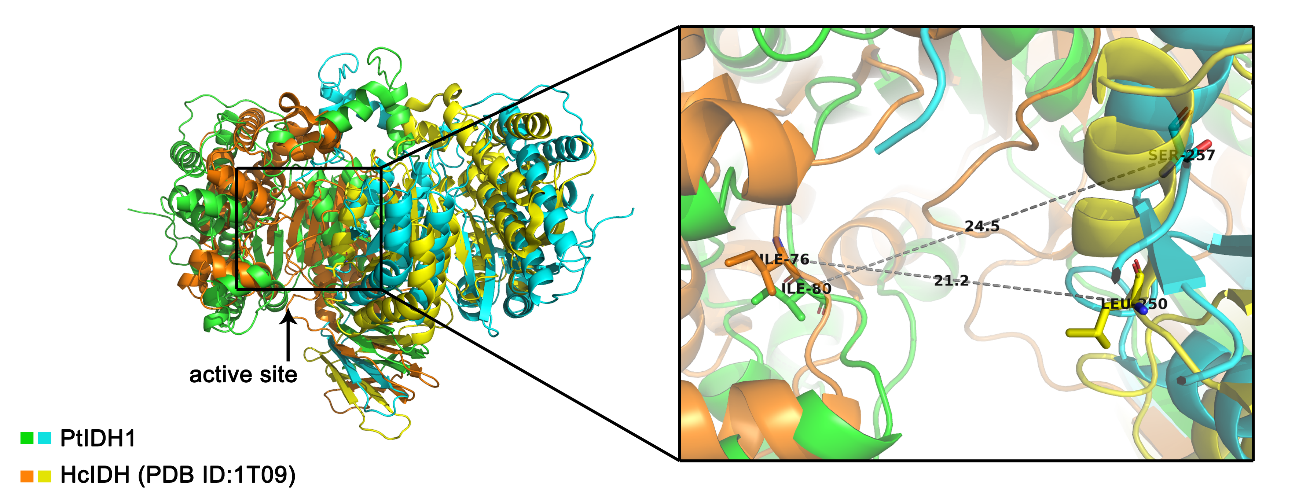


**Figure S5.** Overlay of the dimers of PtIDH1 (green&cyan) and HcIDH (orange&yellow). The enlarged view (right) highlights the different sizes of active sites between PtIDH1 and HcIDH. The distance between the C_α_ -atoms of Ile80 and Ser257 in PtIDH1 and Ile76 and Leu250 in HcIDH are indicated.

**Table S1.** The kinetic parameters of eukaryotic IDHs toward isocitrate.

| **Enzyme** | ***S*_0.5_ (μM)** | **Hill coefficient (*n*_H_)** |
| --- | --- | --- |
| PtIDH1 (Mg^2+^) | 608.1 ± 4.0 | 1.36 ± 0.03 |
| PtIDH1 (Mn^2+^) | 84.9 ± 5.2 | 1.43 ± 0.14 |
| *O. tauri* NAD-IDH (Mg^2+^) [2] | 8.1 | 1.91 |
| *O. tauri* NAD-IDH (Mn^2+^) [2] | 6.6 | 1.83 |
| *C. reinhardti* NAD-IDH (Mn^2+^) [3] | 370 | 1.82 |
| *S. cerevisiae* NAD-IDH (Mg^2+^) [4] | 530 | 3.1 |
| Pea NAD-IDH (Mg^2+^) [5] | 300 | 3.1 |
| Potato NAD-IDH (Mg^2+^) [6] | 690 | 2.5 |

Data are the mean ± SD of at least three independent measurements.

**Table S2.** X-ray data collection and structure refinement statistics.

|  | **PtIDH1_Apo** |
| --- | --- |
| **Data collection** |  |
| X-ray source (Beam line) | SSRF (BL18U1) |
| Wavelength (Å) | 0.97930 |
| Space group | *P* 2_1_ 2_1_ 2_1_ |
| **Cell dimensions** |  |
| a, b, c (Å) | 71.97, 128.55, 124.30 |
| α, β, γ (°) | 90.00, 90.00, 90.00 |
| Resolution (Å) | 47.94-2.71 (2.86-2.71) |
| *R*_merge_ (%) ^a^ | 9.20 (71.60) |
| I/σI | 18.60 (3.80) |
| Completeness (%) | 100.0 (100.0) |
| No. of observed reflections | 422213 (59008) |
| No. of unique reflections | 31982 (4568) |
| Mosaicity (°) | 0.10 |
| Multiplicity | 13.20 (12.90) |
| **Refinement** |  |
| Resolution (Å) | 47.04-2.80 (2.90-2.80) |
| No. of reflections working/test set | 30800/1599 |
| *R*_work_/*R*_free_^b^ | 0.227/0.283 |
| No. of non-hydrogen atoms (protein/water) | 7664/26 |
| **Mean B-factor (Å^2^)** |  |
| overall | 73.88 |
| chain A/B/S | 73.01/74.87/54.97 |
| **RMSD from ideal geometry** |  |
| Bond length (Å) | 0.01 |
| Bond angles (°) | 1.84 |
| **Ramachandran plot** |  |
| Core (%) | 84.0 |
| Allowed (%) | 13.6 |
| Generously allowed (%) | 1.2 |
| Disallowed (%) | 1.2 |
| PDB ID | 6LKZ |

The values in parentheses indicate the highest resolution shell. ^a^*R*_merge_=Σ*_hkl_*Σ*_i_*|*I_hkl_*,*_i_*-<*I*(*_hkl_*)>|/Σ*_hkl_*Σ*_i_I_hkl_*. *R*_pim_=Σ*_hkl_*[1/(n-1)]^1/2^Σ*_i_*|*I_hkl,i_*-<*I*(*_hkl_*)>|/Σ*_hkl_*Σ*_i_* *I_hkl,I_*. ^b^*R*_work_=Σ||F_obs_|-|F_calc_||/Σ|F_obs_|, where F_obs_ and F_calc_ are the observed and calculated structure factor amplitudes. *R*_free_ was calculated same as *R*_work_, using a randomly selected 5% reflections excluded from refinement.

The values in parentheses indicate the highest-resolution shell.

^a^*R*_merge_=Σ*_hkl_*Σ*_i_*|*I_i_*(*hkl*)*_i_*-<*I*(*hkl*)>|/Σ*_hkl_*Σ*_i_I_i_*(*hkl*). ^b^*R*_work_=Σ||F_obs_|-|F_calc_||/Σ|F_obs_|, where F_obs_ and F_calc_ are the observed and calculated structure factor amplitudes. *R*_free_ was calculated same as *R*_work_, using a randomly selected 5% reflections excluded from refinement.

**Table S3.** Primers for amplification of wild-type and mutant PtIDH1 genes.

| **Name ^a^** | **Sequences (5'-3') ^b^** |
| --- | --- |
| PtIDH1_S | GGAATTCCATATGTCCTCCTTGTCAACACTCCGAATC |
| PtIDH1-53_S | GGAATTCCATATGCTCAGTGTCGAGAATAAAGTCGTG |
| PtIDH1-EF_As | CCGCTCGAGCGCGTCGAGGTATCGTTGTAAACGCC |
| PtIDH1_As | CCGCTCGAGCACGTCCGGCTTCTTCTCCTTTTCTTTC |
| PtIDH1_M4A_S | GTAT***gcc***AAG***gct***GGT***gcc***GGT***gct***ATTGACTTTGAAG |
| PtIDH1_M4A_As | CTTCAAAGTCAAT***agc***ACC***ggc***ACC***agc***CTT***ggc***ATAC |
| PtIDH1_M6A_S | GT***gcc***GGT***gct***ATT***gcc***TTTGAA***gca***TTCACTAGAATGC |
| PtIDH1_M6A_As | GCATTCTAGTGAA***tgc***TTCAAA***ggc***AAT***agc***ACC***ggc***AC |
| Mutant_As | CTTCCTTTTTCAATATTATTGAAGCATTTATCAGG |

^a^ “S” and “As”: indicate the sense (S) and antisense (As) primers of the corresponding genes. ^b^ Underlined bases indicate the restriction sites. CATATG, *Nde*I; CTCGAG, *Xho*I. Underlined and bold lower-case bases indicate the mutation site.

References

1. Takahashi, D.; Suzuki, K.; Sakamoto, T.; Iwamoto, T.; Murata, T.; Sakane, F., Crystal structure and calcium-induced conformational changes of diacylglycerol kinase alpha EF-hand domains. *Protein Sci* **2019**, 28, (4), 694-706.
2. Tang, W. G.; Song, P.; Cao, Z. Y.; Wang, P.; Zhu, G. P., A unique homodimeric NAD^+^-linked isocitrate dehydrogenase from the smallest autotrophic eukaryote *Ostreococcus tauri*. *FASEB J* **2015,** 29, (6), 2462-72.
3. Martínez-Rivas, J. M.; Vega, J. M., Studies on the isoforms of isocitrate dehydrogenase from *Chlamydomonas reinhardtii*. *J Plant Physiol* **1994,** 143, (2), 129-134.
4. Lin, A. P.; Demeler, B.; Minard, K. I.; Anderson, S. L.; Schirf, V.; Galaleldeen, A.; McAlister-Henn, L., Construction and analyses of tetrameric forms of yeast NAD^+^-specific isocitrate dehydrogenase. *Biochemistry* **2011,** 50, (2), 230-9.
5. McIntosh, C. A.; Oliver, D. J., NAD-linked isocitrate dehydrogenase: isolation, purification, and characterization of the protein from pea mitochondria. *Plant Physiol* **1992,** 100, (1), 69-75.
6. Tezuka, T.; Laties, G. G., Isolation and characterization of inner membrane-associated and matrix NAD-specific isocitrate dehydrogenase in potato mitochondria. *Plant Physiol* **1983,** 72, (4), 959-63.
