# Supplementary figures and images for: Biochemical Characterization and Crystal Structure of a Novel NAD+-Dependent Isocitrate Dehydrogenase from Phaeodactylum tricornutum
Source: Int J Mol Sci. 2020 Aug 18;21(16):5915. doi: 10.3390/ijms21165915 (PMC7460673; doi:10.3390/ijms21165915)

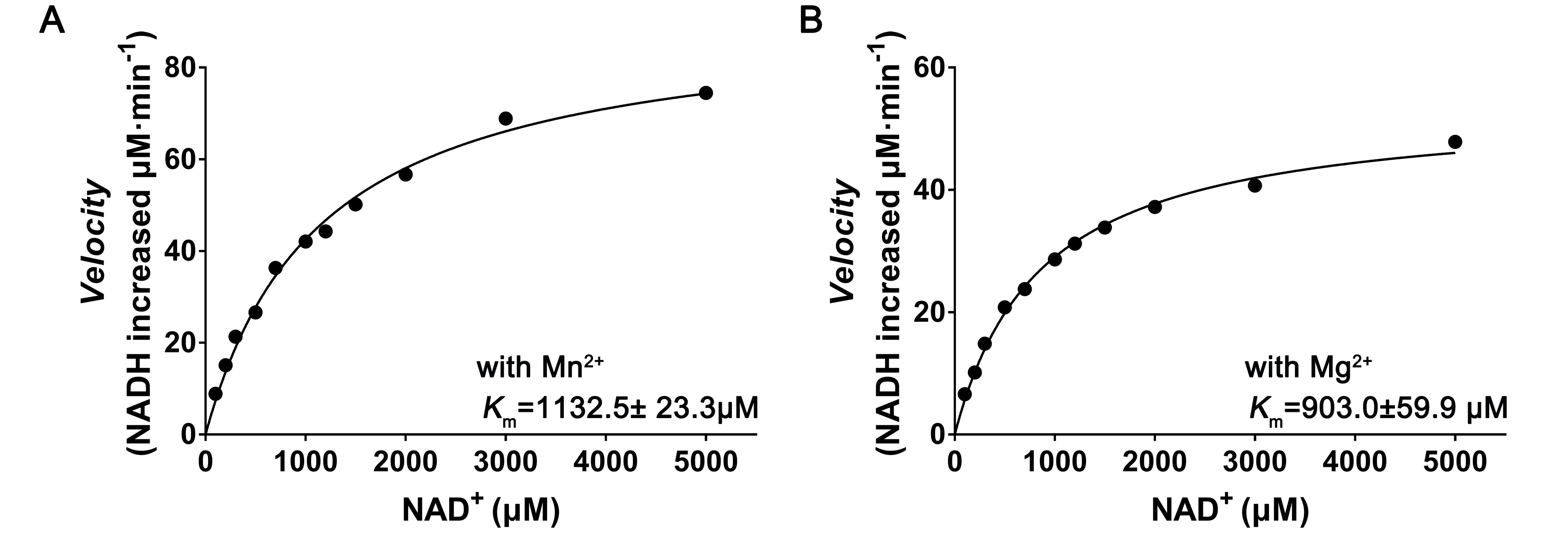

Supplement: Supplementary file 1 [file ijms-21-05915-s001.zip › Supplementary_Proof/Figure_S1_NAD_Km.tif]

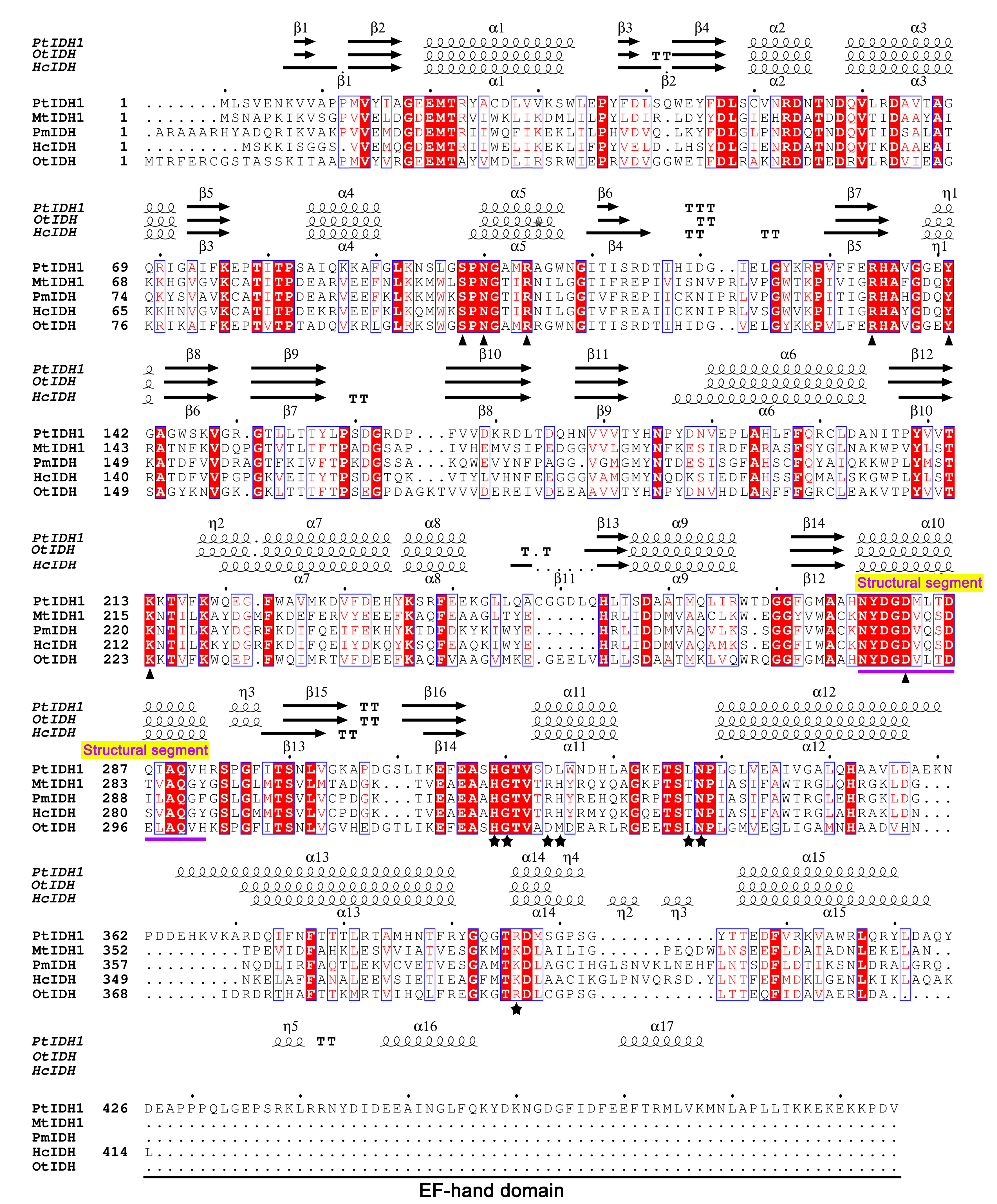

Supplement: Supplementary file 1 [file ijms-21-05915-s001.zip › Supplementary_Proof/Figure_S2_ali.tif]

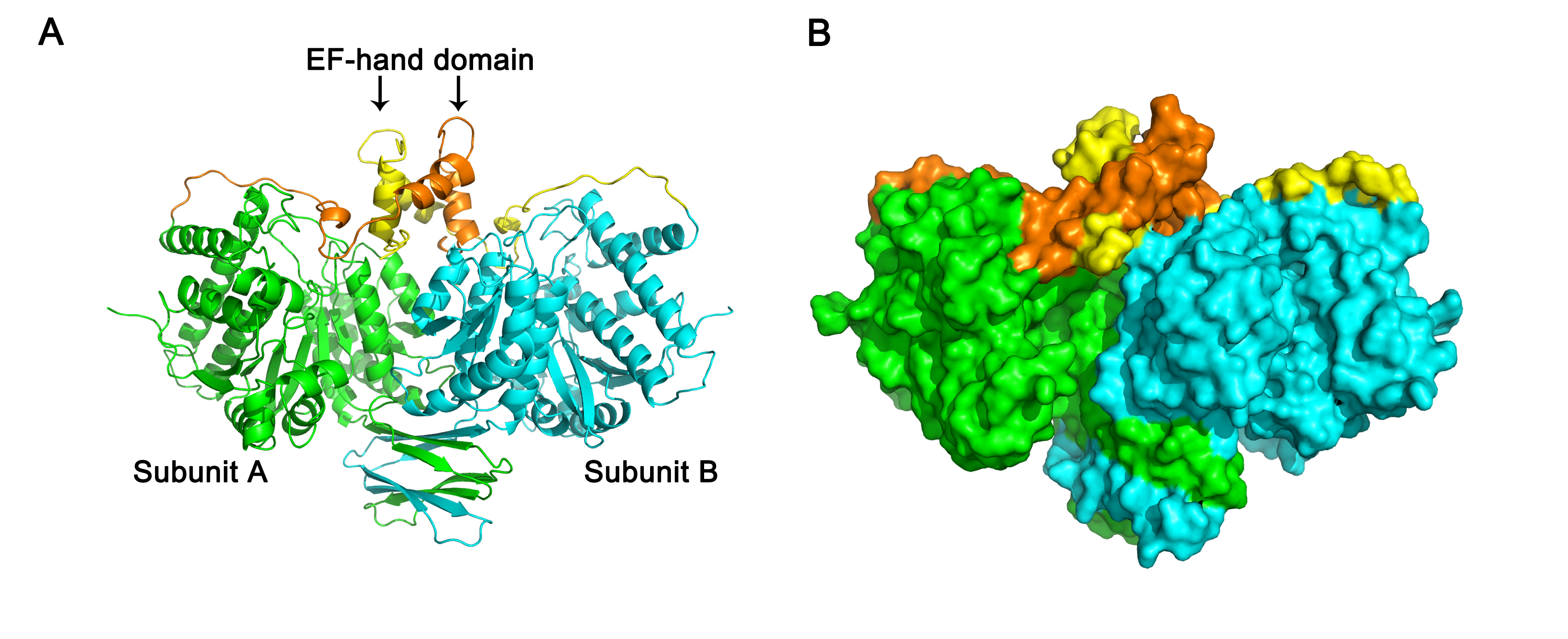

Supplement: Supplementary file 1 [file ijms-21-05915-s001.zip › Supplementary_Proof/Figure_S3_interface.tif]

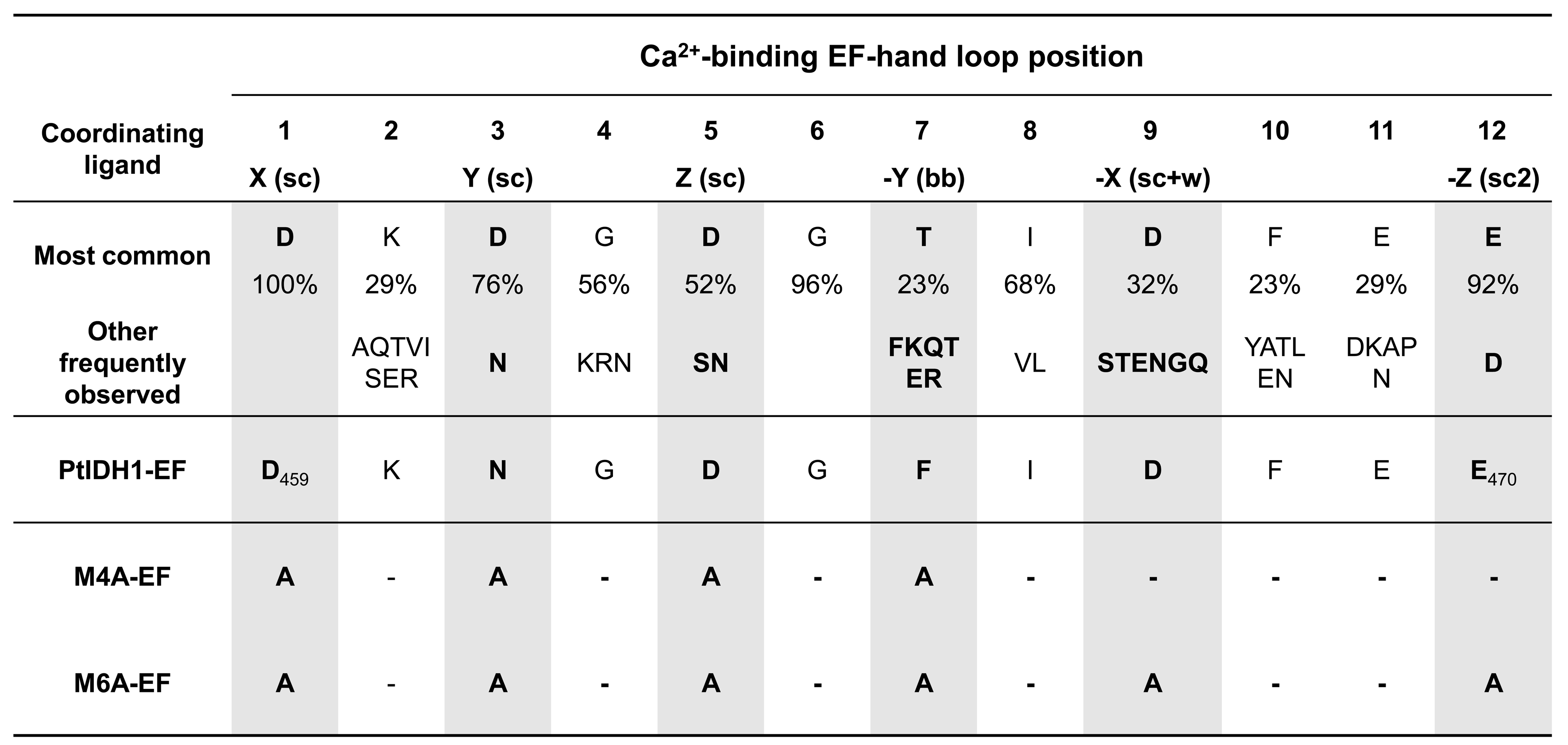

Supplement: Supplementary file 1 [file ijms-21-05915-s001.zip › Supplementary_Proof/Figure_S4_EF.tif]

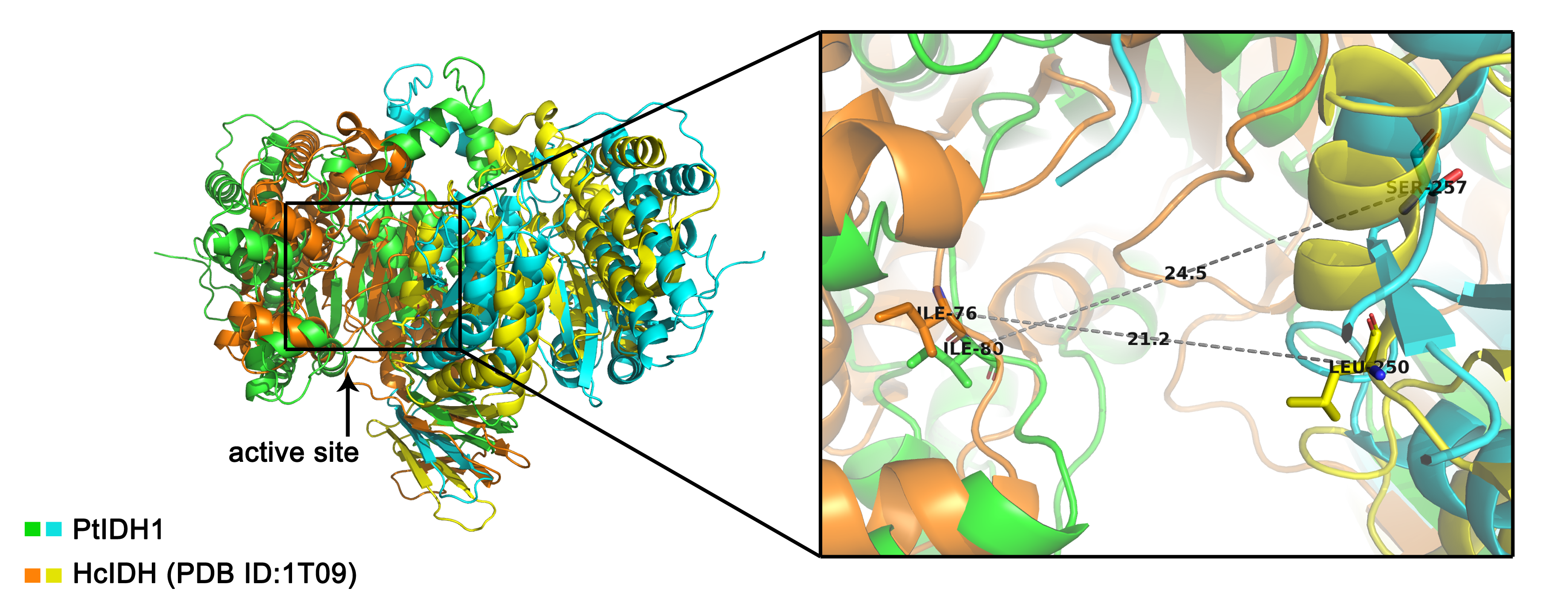

Supplement: Supplementary file 1 [file ijms-21-05915-s001.zip › Supplementary_Proof/Figure_S5_open.tif]
